# Drug-induced dyskinesia in Parkinson's disease. Should success in clinical management be a function of improvement of motor repertoire rather than amplitude of dyskinesia?

**DOI:** 10.1186/1741-7015-11-76

**Published:** 2013-03-20

**Authors:** Jean-François Daneault, Benoit Carignan, Abbas F Sadikot, Michel Panisset, Christian Duval

**Affiliations:** 1 Department of Neurology and Neurosurgery, Montreal Neurological Institute, McGill University, 3801 University street, Montreal, Quebec, H3A 2B4, Canada; 2 Centre de Recherche de l'Institut Universitaire de Gériatrie de Montréal, 4545 Chemin Queen-Mary, Montréal, Québec, H3W 1W4, Canada; 3 Département des Sciences Biologiques, Université du Québec à Montréal, 141 Avenue Président-Kennedy, Montréal, Québec, H2X 1Y4, Canada; 4 Unité des troubles du mouvement André-Barbeau, Centre Hospitalier de l'Université de Montréal, 1560 rue Sherbrooke Est, Montréal, Québec, H2L 4M1, Canada; 5 Département de Kinanthropologie, Université du Québec à Montréal, 141 Avenue Président-Kennedy, Montréal, Québec, H2X 1Y4, Canada

**Keywords:** LID, DID, Levodopa, Deep brain stimulation, DBS, Treatment, Quality of life, Motor complication, Motor fluctuations, Algorithm

## Abstract

**Background:**

Dyskinesia, a major complication in the treatment of Parkinson's disease (PD), can require prolonged monitoring and complex medical management.

**Discussion:**

The current paper proposes a new way to view the management of dyskinesia in an integrated fashion. We suggest that dyskinesia be considered as a factor in a signal-to-noise ratio (SNR) equation where the signal is the voluntary movement and the noise is PD symptomatology, including dyskinesia. The goal of clinicians should be to ensure a high SNR in order to maintain or enhance the motor repertoire of patients. To understand why such an approach would be beneficial, we first review mechanisms of dyskinesia, as well as their impact on the quality of life of patients and on the health-care system. Theoretical and practical bases for the SNR approach are then discussed.

**Summary:**

Clinicians should not only consider the level of motor symptomatology when assessing the efficacy of their treatment strategy, but also breadth of the motor repertoire available to patients.

## Background

Parkinson's disease (PD) is a progressive neurodegenerative disease characterized by a predominant loss of dopaminergic neurons in the substantia nigra pars compacta [1] leading to the development of motor symptoms. Four cardinal motor symptoms are associated with PD: tremor, muscle rigidity, postural instability and akinesia/bradykinesia [2]. PD is also associated with the development of non-motor symptoms stemming from the pathological involvement of particular brain structures and complex neurochemical imbalances [3]. These symptoms include psychiatric manifestations [4], rapid eye movement and other sleep disturbances [5,6], mood disturbance [7,8], bradyphrenia and cognitive deficits [9-12], anosmia [13], fatigue, autonomic system dysfunction and pain [14]. Although both motor and non-motor symptoms can be disabling for patients, current treatments target predominantly the motor dysfunction using mainly dopaminergic therapies. Prolonged use of dopaminergic agents can lead to drug-induced dyskinesia.

Dyskinesia may have deleterious effects on the quality of life of both patients and their caregivers, and create an additional strain on the health-care system. While several approaches are taken by movement disorder specialists to delay or manage dyskinesia, neurologists not specialized in the treatment of movement disorders and general practitioners may find it difficult to control dyskinesia while maintaining clinically significant reductions in typical PD symptoms. In this paper, we propose a novel way to view the clinical management of dyskinesia, which could benefit patient care. In order to comprehend fully the complexity of the problem of dyskinesia, we first provide an overview of the treatments for PD and how they can induce dyskinesia. We then provide a review of the impact of dyskinesia on quality of life and health-care costs.

## Discussion

### How prominent is the problem of PD?

The prevalence rate of PD was estimated a few years ago to be between 100 to 200/100,000 population [15-19], with an incidence rate of 10 to 20/100,000 population [20,21]. However, the number of PD cases is increasing and will have grown from 10 million worldwide in the late 1980s [22] to 40 million in 2020 [23] due mainly to the aging population. While most patients with PD are diagnosed after the age of 55 (see [24,25]), about 10% of patients are diagnosed before the age of forty [26,27] and characterized as 'young-onset PD' [22]. While most young-onset patients exhibit typical parkinsonian symptoms [28], they appear to display slower disease progression [25] and show a tendency for increased prevalence and severity of motor fluctuations and dyskinesia with prolonged _L_-3,4-dihydroxyphenylalanine (L-DOPA) therapy [22,29-32]. Early onset of motor complications may be especially relevant in these patients as they will live with the disease for longer periods [33] with a diminished quality of life [34] and impaired social and economic productivity [34,35].

### What are the current treatments of PD?

Based on the classical model of basal ganglia movement disorders [36-38], the loss of dopaminergic neurons associated with PD results in depletion of dopamine content into the neostriatum. This translates into altered basal ganglia neural activity, producing a change in the output of the basal ganglia-thalamo-cortical pathways. The cardinal hypokinetic symptoms of PD result from a change in the activity of thalamo-cortical inputs to motor cortical areas which impairs voluntary movement [36,39,40]. Consequently, the primary goal of PD treatment is to counteract the depletion of dopamine. Since dopamine causes severe nausea, and cannot easily cross the blood brain barrier, other means of counteracting this dopaminergic deficiency have been developed (see [41] and [42] for comprehensive reviews of current treatment options). In brief, the current gold standard for the treatment of PD motor symptoms is L-DOPA [24,25,41,43-46] associated with a decarboxylase inhibitor such as carbidopa [47-49]. Over the years, several compounds were developed to be used as adjuncts to L-DOPA or as replacement therapy. Catechol-O-methyltransferase (COMT) inhibitors such as entacapone and tolcapone are used as adjuncts to L-DOPA in order to enhance its bioavailability [26,50,51]. Monoamine oxidase-B (MAO-B) inhibitors, on the other hand, are used to extend the duration of action of L-DOPA by decreasing the metabolic degradation of dopamine in the synaptic cleft [1,22,29,46,52-55]. Another class of drugs that can be used as an adjunct or replacement to L-DOPA is dopamine agonists as they bind to dopaminergic receptors, mimicking the action of dopamine. They were initially used to reduce the dose of L-DOPA to control motor complications [24,41] and may be considered for initial monotherapy [56,57], especially in younger patients to delay the onset of dyskinesia.

While medications are the main therapeutic avenue for the alleviation of PD symptoms, surgical procedures can also provide symptomatic relief in some patients. Ablative surgeries have been used in the treatment of motor dysfunction in PD for several decades and can be very effective [58]. Several nuclei of the basal ganglia-thalamo-cortical pathways are targeted using this technique, such as the thalamus [58-69], the globus pallidus internus (GPi) [70-80] and the subthalamic nucleus (STN) [76,81-90]. More recently, deep brain stimulation (DBS) has become an invaluable clinical management tool for medically intractable motor symptoms. Interestingly, DBS targets the same structures that are targeted in ablative surgeries [91]. DBS therapy has the advantage that it is reversible and can be titrated but it suffers from complications and inconveniences related to prosthetic implants [92-98]. In recent years, STN and GPi DBS [95,99-109] have become the targets of choice for effective relief of many motor symptoms associated with PD, including marked reduction of dyskinesia [110,111]. Other structures were recently investigated for the alleviation of specific symptoms [112]. For example, the pedonculopontine nucleus (PPN) [113-116] was targeted for DBS in patients with gait and postural imbalance issues. The centro-median-parafascicular (CM/Pf) complex [117] and the zona incerta [118-121], on the other hand, were targeted in patients with tremor, as an alternative to the well-established thalamic ventrolateral (VL) nucleus. However, whether DBS within these alternative structures has an impact on dyskinesia has yet to be assessed.

Novel and experimental treatments of motor symptoms of PD, some of which are potentially disease-modifying, have also been introduced. One promising avenue is the development of novel drugs for the treatment of PD symptoms. For instance, Adenosine A_2A_-receptor antagonists offer the potential to provide benefits that are not delivered by traditional dopaminergic medications and might avoid dopaminergic side effects through a reduction of the over-activity in the striatopallidal pathway [122]. Many of these drugs are currently in development and are at different phases of clinical trials. Prodrugs are another class of medication currently under development. They are inactive or poorly active compounds that undergo *in vivo *chemical or enzymatic activation that transforms them into an active drug [123]. They have better pharmacokinetic and pharmacodynamic properties than active drugs, thus having the potential of improved oral absorption, stability and passage of the blood brain barrier. For instance, different prodrugs are under development for dopamine, dopamine receptor agonists, better use of the endogenous transport systems of the blood brain barrier as well as different peptide and glutamatergic transport systems [124]. Cell transplant approaches for PD have been considered for several decades with equivocal initial results, especially when compared to currently available treatments. However, recent work has highlighted the potential of this treatment for dopaminergic neuron replacement [125-127]. Finally, there are many potential uses for gene therapy in PD. For example, it can be used to promote the expression of agents which cannot cross the blood-brain barrier, such as neurotrophins [128-131]. Preclinical models using neurotrophic factors provided promising neuroprotective or neuroregenerative outcomes, but initial trials in humans have been mainly disappointing. Gene therapy can also be used to modify the inherent properties of neurons within specific anatomical structures. For example, gene therapy was used to modify the phenotype of STN neurons from predominantly excitatory to predominantly inhibitory in order to restore balance within the basal ganglia-thalamo-cortical network [132-134]. While these are all promising treatments for PD, much work is required with regard to therapy and side effects prior to clinical application to larger patient groups. Relevant to the present paper, it is mainly unknown whether these emerging therapies may delay, treat or worsen dyskinesia.

### What are the main issues with current treatments?

Long-term use of dopamimetic agents, in combination with continued dopaminergic denervation, can generate dyskinesia. Indeed, while dyskinesia are mainly associated with functional alterations within the basal ganglia pathways related to prolonged exposure to L-DOPA, dopamine agonists and DBS can also cause the appearance of dyskinesia [135-138]. The exact mechanism underlying dopamine agonist- or DBS-induced dyskinesia is still under investigation, but it is believed to stem from maladaptive mechanisms related to dopaminergic and glutamatergic systems (see [135] for review). Patients receiving intra-striatal dopaminergic neural grafts can also experience dyskinesia, also without the presence of exogenous dopaminergic agents (off-dyskinesia), possibly due to inappropriate responses to dopamine release by grafted neurons [126,139-141].

There are several different classifications or types of dyskinesia, such as dystonic, ballistic, choreic and myoclonic, which can be monophasic or bi-phasic [142-145], occurring at different times in relation to administration of dopaminergic medication. The most common dyskinesia remain the monophasic choreic type, which are involuntary movements that occur at peak-dose and are considered to be purposeless, non-rhythmic, abrupt, rapid, irregular and un-sustained [143]. We have recently provided the first characterization of the movement patterns of monophasic choreic dyskinesia based on quantitative measures of whole-body movements which highlight their complexity, and variability in amplitude and location over short periods of time [146-150]. This might explain the relative difficulty of patients to control or compensate for their dyskinesia while attempting to either plan or execute everyday motor activities.

Several risk factors are associated with the occurrence of dyskinesia including age of onset of PD [151-154], body weight [155,156], disease duration [157,158], and the level of exposure to L-DOPA [153,159,160]. A necessary factor in the development of dyskinesia appears to be the combination of dopaminergic denervation and long-term exposure to dopamine replacement therapy that promotes changes in the receptor environment and results in an altered clinical response to dopamine [161-164]. Under physiological conditions, striatal and synaptic dopamine levels are maintained at a relatively constant level [165]. The dopaminergic denervation observed in PD, in association with the administration of L-DOPA at intervals during the day, induces oscillations in the concentration of striatal and synaptic dopamine levels [166,167]. This pulsatile stimulation of dopaminergic receptor is associated with functional changes within the basal ganglia [168,169], which results in altered neural activity in the basal ganglia, thalamus and cerebral cortex [115] with associated involuntary movements.

Several fundamental functional alterations in the synaptic environment of the striatum are associated with development of dyskinesia. Dopaminergic denervation-induced pre-synaptic modifications occur at the cellular level which hinders dopamine homeostasis [153,170-172]. In addition, morphological and functional alterations occur in serotoninergic neurons, which may be a homeostatic attempt to counteract the dysregulation in dopamine levels [173]. Changes also occur at the post-synaptic level where dopamine receptor trafficking [158,174], signalling [157] and sensitivity [161,175] are all altered in dyskinetic PD patients. Furthermore, N-methyl-D-aspartate (NMDA), α-amino-3-hydroxy-5-methyl-4-isoxazolepropionic acid (AMPA) [151,152,176,177] as well as metabotropic glutamate receptors [178-181] have been implicated in the maladaptive plasticity associated with dyskinesia (see [135] for review). While the definite mechanisms behind their relative involvement remain to be determined, these receptors are currently being investigated as potential targets for the management of dyskinesia.

Aside from these pre- and post-synaptic changes, other functional and structural changes also play a role in the pathogenesis of dyskinesia. Astrocytes modulate the expression of vascular endothelial growth factor [182], resulting in microvascular remodelling which may be an integral part of the changes in the neural environment that lead to dyskinesia. Over-activity of adenosine A_2A _receptors may also play a role in the generation of dyskinesia [183-188] through facilitation of the striatopallidal pathway [189]. Additionally, modified extracellular concentrations of glutamate [190-193] as well as an altered expression of glutamate transporter genes [191,194,195] have been observed in different basal ganglia structures when dyskinesia are present. Finally, recent studies suggested that degeneration of inter-hemispheric striatal mechanisms may play a significant role in the genesis of dyskinesia through yet undefined mechanisms [196,197]. Taken together, these functional alterations point towards a complex multi-factorial mechanism behind the generation and expression of dyskinesia which could explain why the management of those motor complications is so problematic.

### Why is managing dyskinesia as much art as science?

Due to the complex pathophysiology of dyskinesia, there has been considerable debate about which treatment is more efficacious for best symptom management while still avoiding motor complications [28,32-34,40,44,47,48,51,52,198-201]. Several studies examined the incidence of dyskinesia with different medication (see [41] for an extensive review). Here, we focus on possible treatment options when dyskinesia have already occurred. The primary option for clinicians is to reduce medication dosage; however, this can lead to the resurgence of typical parkinsonian symptoms. The second option is to fragment dosage, reducing each dose and increasing its frequency for more constant delivery as the pulsatile delivery of L-DOPA is, in part, responsible for the observed functional alterations within the basal ganglia. The use of controlled-release oral medication may limit this pulsatile effect [202]. However, the efficacy of such controlled release drugs in treating dyskinesia is investigational at best and there is little evidence to suggest that they may delay the onset of dyskinesia [41]. Nonetheless, the concept behind controlled-release formulations, that is, a more continuous delivery of medication rather than a pulsatile increase in medication normally observed with PD medication, has spurred the development of continuous drug delivery (CDD) systems such as mini-pump guided continuous apomorphine infusion [203], duodenal L-DOPA infusion (Duodopa) [30,201], and transdermal delivery of rotigotine (dopamine agonist) through a patch [204]. Several continuous drug delivery treatments are proposed as useful in reducing the incidence or treatment of dyskinesia [203,205-207], but there is insufficient evidence to characterize them as unequivocally effective [41]. For example, a study on an animal model of PD demonstrated that continuous delivery of rotigotine did not induce dyskinesia and functional sensitization, whereas using an oral formulation at different intervals did [208]. On the other hand, a pilot study on duodenal infusion of L-DOPA was shown to induce similar levels of dyskinesia as pulsatile delivery systems; however, once dyskinesia are present, switching to duodenal L-DOPA reduces the duration of dyskinesia [209]. This highlights the variability in the effectiveness of these treatments. Furthermore, these approaches to dyskinesia treatment are limited due to the complexity of the procedure and the difficult long-term management of patients. Indeed, the invasive nature of some of these treatments limits the number of potential candidates; and the potential for severe complications requires adequate monitoring. Another option is to control dyskinesia by reducing the L-DOPA dose and introducing dopamine agonists. Again, this option is not without problems, including the lower efficacy of dopamine agonists in treating motor symptoms [210-213], as well as increasing the incidence of other disabling side effects such as somnolence, sleep attacks, dizziness, nausea, delusions, impulse control disorders, hallucinations and confusion [214,215]. In addition, one must keep in mind that some studies have observed the appearance of dyskinesia with the use of dopamine agonists without the concomitant presence of L-DOPA [213].

There are currently very limited direct drug treatments for dyskinesia as only two medications were shown to be efficacious: amantadine and clozapine [41]. Amantadine is a NMDA receptor antagonist [216] that was shown to reduce significantly the duration and severity of dyskinesia in several studies [216-218]. However, its mechanism of action leading to reduction in dyskinesia has yet to be conclusively determined. Clozapine is a high affinity serotoninergic agonist as well as a low affinity dopamine agonist [219-221]. One study demonstrated the ability of clozapine to reduce dyskinesia significantly [222]. However, the severe side effects associated with clozapine, such as agranulocytosis [223], central nervous system depression, seizures, dementia, and myocarditis [224], limit its use in clinical practice as it requires strict monitoring.

Surgical interventions can also reduce dyskinesia in a subset of patients as both STN and GPi DBS were shown to reduce dyskinesia effectively [103,109]. One possible mechanism behind the reduction in dyskinesia is reduction in medication dose following surgical treatment [225]. However, the end result is highly dependent on several factors such as lead placement, stimulation parameters and level of reduction in medications. Another surgical intervention that has demonstrated a reduction in dyskinesia is pallidotomy [73,74,226]. In fact, this intervention was shown to be as effective as STN DBS for the reduction of dyskinesia [74]. Again, the outcome of this procedure is greatly dependent on lesion extent and location.

Future avenues for drug treatment of dyskinesia include the development of adenosine A_2A _receptor antagonists [227,228] as well as the use of metabotropic glutamate receptor 5 (mGluR5) antagonists [229] and orthosteric metabotropic glutamate receptor 4 (mGluR4) agonists [230]. While these compounds are currently in different testing phases, a few studies using adenosine A_2A _receptor antagonists and mGluR5 antagonists have demonstrated a significant reduction in dyskinesia induction in animal models [183,186,231] and subgroups of human samples [227,229,232]. On the other hand, orthosteric mGluR4 agonists are only beginning to be studied for their effect on the indirect pathway of the basal ganglia.

Maintaining therapeutic efficacy while at the same time trying to control dyskinesia can be difficult with all treatments for PD. Clinicians often progressively introduce an intricate combination of medications that could help re-establish neurotransmitter balance and avoid motor fluctuations. Unfortunately, the unavoidable dopaminergic denervation and receptor imbalances render this task increasingly difficult as the disease progresses.

### How prominent is the problem of dyskinesia and its management?

The incidence of dyskinesia is estimated at 30% to 50% after five years of initiating L-DOPA treatment [142,198]. As the disease progresses, the incidence can increase to 60% to 100% after 10 years [65,198,211,233,234]. These figures are even higher in young-onset PD where it is observed that almost all patients experience dyskinesia after only six years of treatment [22]. Once these motor fluctuations occur, increased monitoring of patients is required. However, the lack of movement disorders specialists able to handle such complex side effects of medication hinders proper monitoring of these patients. In the United States, the ratio of neurologists varies drastically between regions ranging from a low of 1 and a high of 11/100 000 population [235] with an average close to 5/100 000 population [236]. In Canada, in 2008, the number of neurologists varied between 0 and 3/100 000 population in different regions of a geographically vast country [237]. While most European countries fare relatively well with an average of 5 neurologists per 100 000 population [236], Asia, where the majority of the world's population resides and where the expected number of PD cases is expected to grow several fold in upcoming years [238], is in dire need of neurologists with less than 1/100 000 population [236]. Of note is that these figures encompass all neurologists; the number of movement disorders specialists, who possess the necessary tools to adequately manage the symptoms of PD and motor complications associated with their treatment, is much lower, and to our knowledge, has never been evaluated. Another issue facing patients with motor fluctuations is that most movement disorders specialists are located in larger cities; thus forcing patients from remote communities to travel great distances for medical consultations and follow-ups. These issues may explain why only 45% of patients with PD in Ontario (Canada) have access to a specialist at least once a year [239]. The lack of access to trained clinicians has a negative impact on patient care since constant management of medication is required to delay or negate the undesired motor fluctuations.

### What would be the impact of better management of dyskinesia on quality of life?

The ability to engage and maintain social interactions is inevitably linked to the ability to interact with the physical environment and, as such, is associated on the level of independence of patients. In patients with PD, reduced participation in social activities appears in part related to loss of mobility and impairs quality of life [240,241]. This phenomenon is later exacerbated due to disease progression and complications related to treatments [242,243]. However, the actual impact of dyskinesia on quality of life is still controversial. Some researchers have suggested that dyskinesia have only a moderate impact on quality of life of patients [198,244-246]. One study even observed an improvement in quality of life in PD patients with dyskinesia [244]. Another recent study demonstrated that 'Patients with PD experiencing dyskinesia are less likely to be concerned about dyskinesia and more likely to prefer dyskinesia over parkinsonian symptoms compared to patients without dyskinesia' [247]. This may be explained by the patient's own perspective on the impact of dyskinesia on his/her motor repertoire, that is, the movements a particular patient deems important for his/her quality of life. Of course, if dyskinesia have a moderate impact on the motor repertoire, it is likely that he/she will not consider dyskinesia as problematic. Patients would rather be able to perform their activities than be constricted by their parkinsonian symptoms. However, such findings must be interpreted carefully, in light of recent evidence showing that dyskinetic patients may suffer from anosognosia, that is unawareness of deficits associated with an illness [248]. Accordingly, even if they do not complain about their involuntary movements, dyskinesia may still have a deleterious effect on their motor repertoire. As such, mild dyskinesia themselves may not be problematic, but more severe forms may reduce quality of life by impacting on the patients' motor repertoire.

In fact, other studies showed that the presence of dyskinesia is a key factor in determining the quality of life of patients [249-251], especially in young patients who participate in the workforce. Studies showed that the main dimensions of quality of life that are affected by dyskinesia are psychological, social [252,253] and stigma [253-255]. This may be the result of loss in mobility, increased falls [256], weight loss [156] and even modifications of motor behavior in the OFF state [257]. Other studies demonstrated that the reduction in quality of life of PD patients with dyskinesia [258-260] could also be a result of higher levels of anxiety [261-264] and depression[260], more so than in patients without dyskinesia. However, in the study of Montel *et al. *[253], the only factor that had a significant impact on quality of life was the presence of dyskinesia, not neuropsychiatric manifestations. This indicates that dyskinesia can affect patient quality of life directly and also by inducing, or at least modulating the level of different neuropsychiatric disorders.

The impact of dyskinesia on the quality of life of PD patients can also be evaluated by assessing the effectiveness of interventions aimed at controlling dyskinesia on quality of life. For instance, a recent study demonstrated that PD patients had a significant improvement in quality of life after 18 months of continuous intra-duodenal L-DOPA infusion [265]. Interestingly, they did not observe a significant change in 'ON medication' motor symptomatology after treatment but did observe a significant reduction in dyskinesia. As such, the reduction in dyskinesia may have played a role in the improvement of quality of life. Similar results were obtained in patients undergoing GPi DBS where the reduction in dyskinesia scores was highly correlated with the improvement in overall quality of life [266]. While these are merely two examples of studies using quality of life as primary or secondary endpoints to assess the impact of different interventions, it is becoming more common to use quality of life to evaluate therapeutic effectiveness.

Another issue to consider is that dyskinesia also impact upon on the quality of life of patients' primary caregivers (for example, spouses). Indeed, as the disease progresses and patients with PD begin dealing with a loss of independence, the quality of life of their caregiver also degrades as they are more prone to social isolation, psychological problems, such as depression, and physical issues [267-270]. This is evident through the results of McCabe *et al. *[271] where PD patients and caregivers only differed in physical- and psychological-related quality of life. Social interaction and environmental quality of life scores were not significantly different [271]. These issues become more prominent with disease progression when motor complications, such as dyskinesia, are apparent [272]. Importantly, it has been demonstrated that psychosocial factors such as social support are critically important to the caregivers' quality of life [273]. As health-care systems are over-extended and promote the implementation of community care programs as a means of alleviating pressure on the system, the capacity of caregivers to provide support becomes essential [274]. If caregiver burden is excessive, it may reduce the quality of the care patients require [273]. As such, it is important to acknowledge and find ways to optimize the caregivers' quality of life.

### What would be the impact of better management of dyskinesia on the health-care system?

As the disease progresses, so does the burden on patients and the health community [83,275]. Studies have demonstrated the immense effect of dyskinesia on the costs of treating PD patients. For instance, a European study showed that the average cost per annum for the treatment of PD patients without dyskinesia was €11,412, but it more than doubled to €24,990 in patients with severe dyskinesia [260]. This increase in treatment cost was accounted for by both non-medical expenditures, such as community services and unpaid help provided by the caregiver, and medical expenditures related to medication and hospitalization due to more complex and expensive treatment regimens [260]. A French study also demonstrated that the presence of dyskinesia more than doubled treatment costs and increased medical visits [276]. They also observed that the severity of dyskinesia increased medical costs by increasing the need for caregivers. This led them to estimate the total annual medical cost of dyskinesia in France to be between 588 and 812 million francs [276]. Furthermore, a recent study from the United States showed that dyskinesia resulted in an increase in total treatment costs by 29%, and PD-related treatment costs by 78% compared to costs incurred by PD patients without dyskinesia [277]. This translates into an increase of $5,549 in the year following the first appearance of dyskinesia when compared to PD patients without dyskinesia. The majority of this amount was related to an increase in PD-related costs of $4,456 in patients with dyskinesia; not to costs associated with co-mobidities [277].

A major problem is that these direct costs have to be added to the already increased health-related expenditures associated with having PD compared to healthy aging [278]. In Canada, the annual direct costs related to PD were estimated at $202 million, which includes hospital (44%), drugs (49%) and physician consultations (7%). Indirect costs associated with mortality (38%) and morbidity (62%) were estimated at $245 million, for a total of approximately $447 million [278]. Interestingly, a great proportion of indirect costs are related to early retirement. The direct health-care cost of PD in the United States was estimated at $10,349 per patient per year [279]. Combining these direct costs with estimates of indirect costs, the total costs of PD in the United States may be as high as $23 billion annually [279]. If we consider that the number of persons 65 years of age and older is expected to increase significantly over the upcoming years, the cost of treating PD patients is likely to exceed $50 billion annually in the United States by 2040 [279]. In China, the problem is even greater because of the larger number of patients. In 2004, it was estimated that the yearly health-care cost was about $925 per patient, which represents half of the mean individual annual income [280], for a total of $1.57 billion annually. The total cost correlated significantly with disease severity and the frequency of outpatient visits [280]. It is clear that better patient management is required and one approach is to develop and implement evidence-based practice. The question then becomes if the reduction of dyskinesia incidence and severity can modulate the costs. A recent study examined the effectiveness (time to levodopa and time to levodopa-induced dyskinesia), cost, and quality-adjusted life-years in two trials of dopamine agonists. They showed that rasagiline delayed the onset of dyskinesia by 10% and reduced costs by 18% per patient over five years [281]. Furthermore, a French study estimated that each 10% of reduction in OFF periods would result in a 5% reduction of direct medical costs [282]. These studies demonstrate that finding approaches to control either the incidence or the severity of dyskinesia and other motor fluctuations should be developed and implemented in order to reduce the burden on the health-care system.

### What is the theory behind our proposed approach to the treatment of dyskinesia?

Evidence-based practice aims to apply the best available evidence from scientific investigations to clinical decision making. To apply evidence-based practice for the management of dyskinesia, information about the influence of dyskinesia on voluntary movements must be known so as to understand the challenges facing patients when planning and executing movements from their motor repertoire. It is important to discriminate between activities of daily living and motor repertoire of patients as activities of daily living are essential for minimal functional independence while the motor repertoire encompasses all movements deemed important for a good quality of life for a specific patient. As such, the motor repertoire will be personalized and will vary greatly depending on the movements patients wish to perform on a regular basis. Finally, it is important to assess whether other symptoms are concomitantly present with dyskinesia; which may in fact be responsible for motor deficits. To date, several algorithms have been proposed to manage dyskinesia [283,284]. Interestingly, these algorithms are geared towards markedly reducing or eliminating dyskinesia, without necessarily taking into account how the proposed strategy affects the motor repertoire of patients. This is important since some patients may rather have mild dyskinesia then undergo the process of medication change, especially if dyskinesia do not hinder their motor repertoire. Indeed, the reduction in dyskinesia through either a reduction in medication dosage or a change in medication could lead to a resurgence of typical hypo- or hyper-kinetic parkinsonian symptoms impeding the patient's voluntary motor behaviors and hence reduce his quality of life for that specific period. The clinician will judge whether the reduction in dyskinesia following treatment regimen modification based on these algorithms is clinically satisfactory. For this, clinicians rely mostly on their experience and patient feedback. They can also use clinical scales [285-288] to assess the amplitude of dyskinesia and their impact on activities of daily living. However, current scales only provide a general sense of the amplitude of dyskinesia and their impact. Most do not measure the impact of the amplitude of dyskinesia on voluntary movements and certainly not on the entire motor repertoire of patients. In fact, a recent review of the different scales for the assessment of dyskinesia found that of the eight scales used in PD, only two were recommended for use (that is, the Abnormal Involuntary Movement Scale (AIMS), and the Rush Dyskinesia scale) [288]. The AIMS assesses the amplitude of dyskinesia in each limb whereas the Rush also incorporates a section on the impact of dyskinesia on certain activities of daily living such as putting on a coat. A recent scale, the PDYS-26, a patient-based questionnaire, focuses solely on the impact of dyskinesia on activities of daily living [289]. One main issue of these scales is that they cannot segregate the impact of dyskinesia and cardinal symptoms of PD on the performance of motor behaviors. Another point that requires attention is that, as mentioned above, activities of daily living do not circumscribe the whole motor repertoire deemed necessary by each patient; they merely represent general tasks that provide some functional independence. For example, a patient who is an artist painter with low amplitude dyskinesia may deem that his/her dyskinesia are devastating, while most daily living activities are actually intact (that is, he can put on a coat, cut his food and dress himself but, he cannot perform the fine voluntary movements required for him to paint a canvas). Then, one could legitimately ask the following question: how does the amplitude of dyskinesia relate to its impact on voluntary movements performed in daily life? The opposite could also be true. A patient with high levels of dyskinesia may judge that his/her involuntary movements are not an issue since they prefer to be dyskinetic rather than OFF, as proposed in a recent paper [247].

We propose that the evaluation of the impact of dyskinesia be viewed as a function of a signal-to-noise ratio (SNR). The concept of the SNR is based on the fact that success of voluntary movements (the motor output) is directly correlated to the magnitude of the intended voluntary movement (the signal) and inversely correlated with the magnitude of the involuntary movement (the noise) in the motor stream [290-297]. In other words, the likelihood of success in performing voluntary movements is not only dependent on the magnitude of the symptoms present, but also dependent on the type of movement performed by the patients. Such an analysis would make it possible to determine the motor repertoire available to patients based on the magnitude of symptoms. For instance, if a patient presents only with tremor, the SNR could be represented by equation 1:

$$\frac{\left[\text{Voluntary}\;\text{drive}\;\text{for}\;\text{a}\;\text{specific}\;\text{movement}\right]}{\left[\text{Tremor}\right]}=\text{Motor}\;\text{output}$$

Here, tremor would become deleterious only if the intended movement is below a threshold that will allow tremor to be close to, or supersede, the voluntary movement in amplitude. It could also be deleterious if the frequency of the intended movement is close to the frequency of that tremor [298-300]. Of course, this is an oversimplification as PD patients rarely exhibit only one motor symptom. Therefore, a more accurate representation of the SNR observed in PD patients would be equation 2:

$$\frac{\left[\text{Voluntary}\;\text{drive}\;\text{for}\;\text{a}\;\text{specific}\;\text{movement}\right]}{\left[\text{Tremor}\right]+\left[\text{Bradykinesia}\right]+\left[\text{Rigidity}\right]+\left[\text{Postural}\;\text{Instability}\right]}=\text{Motor}\;\text{output}$$

Here, the noise would be the sum of all cardinal motor symptoms, regardless of their neural origin. Indeed, bradykinesia could be caused by bradyphrenia during complex decision making, rather than a lack of cortical activation by thalamo-cortical pathways. Interestingly, as the disease progresses and motor complications arise, more 'noise' parameters could be added to equation 2 such that dyskinesia could be taken into account (equation 3):

$$\frac{\left[\text{Voluntary}\;\text{drive}\;\text{for}\;\text{a}\;\text{specific}\;\text{movement}\right]}{\left[\text{Tremor}\right]+\left[\text{Bradykinesia}\right]+\left[\text{Rigidity}\right]+\left[\text{Postural}\;\text{Instability}\right]+\left[\text{Dyskinesia}\right]}=\text{Motor}\;\text{output}$$

**Figure 1:**
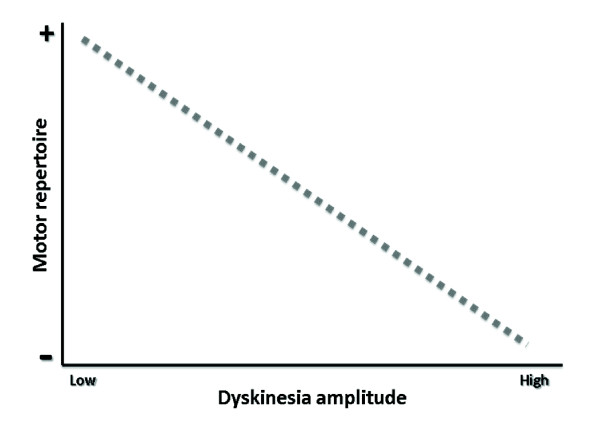
**Shown here is the theoretical relationship between the amplitude of involuntary movements (dyskinesia) and the motor repertoire of patients**. We hypothesize that higher amplitudes of dyskinesia will result in lower signal-to-noise ratio (SNR; dashed line) and, therefore, a loss of motor repertoire.

Success for a particular task would be predicated upon the ratio between the amplitude of the intended movement (the signal; the numerator) and the magnitude of symptoms (noise; the denominator) (see Figure 1).

This relationship between voluntary and involuntary movements was demonstrated by us in previous work [290-297]. For instance, we showed that during slow alternating movements at the wrist, tremor was detected [295], and its amplitude was directly correlated with deficits of accuracy [294]. During fast movement, tremor was undetected, and its amplitude previously assessed in the postural condition was unrelated to performance [294,297]. Furthermore, we showed that ventro-lateral thalamotomy [59,61,294] had no impact on fast movements, but increased the SNR by removing tremor, hence improving tremendously the accuracy during slow movements [294]. We also showed that in tasks where the voluntary movement was performed with varying amplitude and velocity, the faster sections presented with higher SNR, and there was a reduction in deviation from the intended trajectory of the movement [294,295]. Accordingly, the amplitude of velocity of the intended movement seemed to be important in determining the impact of involuntary movements on voluntary motor acts. This concept relates to Fitts law [301], which proposes that two movements having the same amplitude may possess different velocity profiles, depending on the difficulty (target size) of the task. For example, bringing a glass of water to the mouth may have the same amplitude as bringing a spoon full of soup, but the velocity will not be the same because of the increased difficulty associated with keeping the soup in the spoon. As such, in order to properly assess the complexity of a voluntary movement, both its amplitude and velocity must be examined. In patients where whole-body peak-dose dyskinesia were recorded simultaneously with voluntary movements (same tasks as above), we found that during fast hand movements, dyskinesia were not visible [296]. Interestingly, patients with dyskinesia presented with levels of bradykinesia similar to those of PD patients without dyskinesia [296]. We also found no relationship between the level of dyskinesia and accuracy during slow movements [293], indicating that dyskinesia may not have been the primary source of error during movements that required accuracy. This strongly supports the concept that 'noise' is not limited to visible involuntary movements, but may also include other symptoms such as rigidity or bradykinesia [291] as proposed in equations 2 and 3. In the aforementioned study, patients had little or no clinically-detectable rigidity, so bradykinesia was probably the main cause of reduction in motor performance. Taken together, this illustrates that different types of noise observed in PD can be independent from each other at the neurophysiological level but can each contribute to the performance of a given task. In another study, we demonstrated that patients with Huntington's disease presenting with chorea were not impaired during fast hand movements. However, they presented with large errors during slow manual tracking, which correlated with the amplitude of chorea. This illustrates again that involuntary movements can be of no consequence when the SNR is large enough. This also indicates that the SNR concept could be applied to pathologies other than PD.

**Figure 2:**
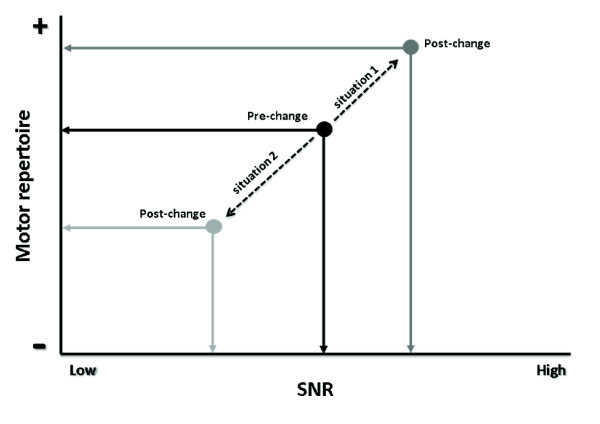
**Two examples to illustrate opposite results following drug regimen change**. In situation 1, a change in drug regimen decreased dyskinesia amplitude which then led to increased signal-to-noise ratio (SNR) (dark grey lines), and consequently increased motor repertoire. In situation 2, the same change in drug regimen also led to a reduction of dyskinesia amplitude. However, there is resurgence of typical motor symptoms associated with PD, thus increasing the noise, which will induce a decrease of overall SNR, hence a reduction in the motor repertoire (light grey lines). Here, the patient did not benefit from the reduction of dyskinesia, as his/her motor repertoire worsened. These examples illustrate the challenges faced by clinicians when managing dyskinesia. PD, Parkinson's disease.

The aforementioned data on PD fits well with issues facing clinicians. Indeed, any reduction in dyskinesia levels could lead to increased typical parkinsonian motor symptoms. Accordingly, clinicians may be replacing one kind of noise with another one (this concept is illustrated in Figure 2).

To better illustrate this theory, we present below two hypothetical situations that could be encountered in clinical practice (Figure 2).

Situation 1: the clinician reduces L-DOPA or dopamine agonist dosage and the level of dyskinesia is reduced. This results in an increased motor repertoire because of the increased SNR. Dyskinesia management is effective and should be pursued.

Situation 2: the clinician reduces medication dosage and the level of dyskinesia is reduced, but leads to a reduction in motor repertoire. As such, the dyskinesia portion of the noise is reduced but is accompanied by an increase in noise associated with typical parkinsonian symptoms present when medication is lacking, such as bradykinesia or rigidity. Here, the treatment regimen should be modified until situation 1 is achieved. If situation 1 cannot be achieved, it may be that having some dyskinesia is the preferred solution since the motor repertoire is greater with dyskinesia, as discussed by our group [293,296] and others [302]. Surgery may be considered as an alternative in this case because, as mentioned above, it may control dyskinesia possibly through a reduction in medication. The aforementioned approach would seem logical to movement disorders specialists, but may be more difficult to implement by less experienced clinicians treating patients with PD experiencing motor fluctuations.

Accordingly, we propose that there is a SNR related to dyskinesia below which the execution of a voluntary movement is rendered impossible (or not functionally possible). Whether this SNR is systematic across patients or specific to each patient is currently under investigation in our laboratory. We also propose that a reduction of dyskinesia amplitude through a proper medication regimen modification will result in an increased motor repertoire only if typical parkinsonian symptoms do not re-emerge to levels affecting significantly the SNR for specific tasks.

### How may this strategy be translated into clinical practice?

We propose that clinicians may be able to view treatment success as an optimization of each patient's motor repertoire, rather than simply targeting symptomatology. Highly-trained movement disorders specialists probably use such an approach intuitively, but it is the lack of tools to help clinicians less experienced in dealing with PD patients that should be addressed. For instance, the presence of dyskinesia should be deemed detrimental if it significantly impacts the SNR and thus the motor repertoire of each patient. Based on the aforementioned evidence, there is a need to develop clinical evaluation protocols that specifically assess the motor repertoire of patients. Such a tool must reflect the wide range of movements performed during everyday life activities, it must incorporate a customizable section and be easy to perform, as well as give clinicians the ability to follow the progression within patients and compare the results between patients. While acknowledging that current clinical scales for the evaluation of dyskinesia provide invaluable information regarding their amplitude and impact on some activities of daily living, they lack the specificity for evaluating the range of the motor repertoire accessible to patients. We understand the immense difficulties associated with the development of a clinical scale of this type but, using such an evaluation, the clinician would be in a better position to determine whether the intervention was helpful to the patient, regardless of its effect on symptomatology. We are currently in the process of assessing the motor repertoire of patients without dyskinesia and with different levels of dyskinesia in order to develop a model of interaction between symptomatology and motor behaviors. Once this relationship is known, the development of such a tool could be envisioned.

## Summary

The treatment of PD requires the evaluation of several motor symptoms affecting the quality of life of patients. The limited number of movement disorders specialists and the increasing number of patients with PD places a toll on health-care systems world-wide. The need to develop and implement evidence-based medicine is urgent. In this review, we proposed a novel way to view the clinical management of motor symptoms in PD and more specifically of dyskinesia. While we acknowledge that this view requires further testing, we believe that systematizing the approach to the treatment of motor symptoms in PD will lead to an improvement in patient quality of life and, hopefully, a relief on our health-care system.

## Abbreviations

AIMS: Abnormal Involuntary Movement Scale; AMPA: α-amino-3-hydroxy-5-methyl-4-isoxazolepropionic acid; CDD: continuous drug delivery; CM/pF: centro-median-parafascicular complex; COMT: catechol-O-methyl tttransferase; DBS: deep brain stimulation; GPi: globus pallidus internus; L-DOPA: _L_-3,4-dihydroxyphenylalanine; MAO-B: monoamine oxydase B; mGluR4: metabotropic glutamate receptor 4; mGluR5: metabotropic glutamate receptor 5; NMDA: N-methyl-D-aspartate; PD: Parkinson's disease; PPN: pedonculopontine nucleus; SNR: signal-to-noise ratio; STN: subthalamic nucleus; VL thalamus: ventrolateral thalamus.

## Competing interests

JFD, BC, AFS and CD declare that they have no competing interests. MP has received research grants from Teva Neuroscience, Novartis, and Allergan. He has been a lecturer for Allergan, Merz, Novartis and Teva. He has participated in advisory boards for Merck, EMD Serono, Allergan, Merz, Novartis, and Teva.

## Authors' contributions

JFD, BC and CD contributed to the design and content of the manuscript. AFS and MP contributed to the content of the manuscript. All authors contributed to revisions and approved the final version of the manuscript.

## Pre-publication history

The pre-publication history for this paper can be accessed here:

http://www.biomedcentral.com/1741-7015/11/76/prepub

## References

[B1] Ehringer H Hornykiewicz O [Distribution of noradrenaline and dopamine (3-hydroxytyramine) in the human brain and their behavior in diseases of the extrapyramidal system] Klin Wochenschr 1960 38 1236 1239 13726012 10.1007/BF01485901

[B2] Jankovic J Parkinson's disease: clinical features and diagnosis J Neurol Neurosurg Psychiatry 2008 79 4 368 376 18344392 10.1136/jnnp.2007.131045

[B3] Zgaljardic DJ Foldi NS Borod JC Cognitive and behavioral dysfunction in Parkinson's disease: neurochemical and clinicopathological contributions J Neural Transm 2004 111 1287 1301 15480839 10.1007/s00702-004-0178-z

[B4] Gallagher DA Schrag A Psychosis, apathy, depression and anxiety in Parkinson's disease Neurobiol Dis 2012 46 581 589 22245219 10.1016/j.nbd.2011.12.041

[B5] Gagnon JF Postuma RB Mazza S Doyon J Montplaisir J Rapid-eye-movement sleep behaviour disorder and neurodegenerative diseases Lancet Neurol 2006 5 424 432 16632313 10.1016/S1474-4422(06)70441-0

[B6] Postuma RB Gagnon JF Vendette M Charland K Montplaisir J Manifestations of Parkinson disease differ in association with REM sleep behavior disorder Mov Disord 2008 23 1665 1672 18709686 10.1002/mds.22099

[B7] Tan LC Mood disorders in Parkinson's disease Parkinsonism Relat Disord 2012 18 Suppl 1 S74 76 22166461 10.1016/S1353-8020(11)70024-4

[B8] Hemmerle AM Herman JP Seroogy KB Stress, depression and Parkinson's disease Exp Neurol 2012 233 79 86 22001159 10.1016/j.expneurol.2011.09.035 PMC3268878

[B9] Peavy GM Mild cognitive deficits in Parkinson disease: where there is bradykinesia, there is bradyphrenia Neurology 2010 75 1038 1039 20855847 10.1212/WNL.0b013e3181f39d35

[B10] Perez Trullen JM Modrego Pardo PJ Vazquez Andre ML Bradyphrenia and parkinsonism Age Ageing 1994 23 524 9231949 10.1093/ageing/23.6.524

[B11] Rogers D Bradyphrenia in parkinsonism: a historical review Psychol Med 1986 16 257 265 3523569 10.1017/s0033291700009077

[B12] Rogers D Bradyphrenia in Parkinson's disease Br J Hosp Med 1988 39 128 130 3359080

[B13] Doty RL Olfaction in Parkinson's disease and related disorders Neurobiol Dis 2012 46 527 552 22192366 10.1016/j.nbd.2011.10.026 PMC3429117

[B14] Del Sorbo F Albanese A Clinical management of pain and fatigue in Parkinson's disease Parkinsonism Relat Disord 2012 18 Suppl 1 S233 236 22166444 10.1016/S1353-8020(11)70071-2

[B15] de Rijk MC Breteler MM Graveland GA Ott A Grobbee DE van der Meche FG Hofman A Prevalence of Parkinson's disease in the elderly: the Rotterdam Study Neurology 1995 45 2143 2146 8848182 10.1212/wnl.45.12.2143

[B16] de Rijk MC Launer LJ Berger K Breteler MM Dartigues JF Baldereschi M Fratiglioni L Lobo A Martinez-Lage J Trenkwalder C Hofman A Prevalence of Parkinson's disease in Europe: a collaborative study of population-based cohorts. Neurologic Diseases in the Elderly Research Group Neurology 2000 54 Suppl 5 S21 23 10854357

[B17] de Rijk MC Tzourio C Breteler MM Dartigues JF Amaducci L Lopez-Pousa S Manubens-Bertran JM Alperovitch A Rocca WA Prevalence of parkinsonism and Parkinson's disease in Europe: the EUROPARKINSON Collaborative Study. European Community Concerted Action on the Epidemiology of Parkinson's disease J Neurol Neurosurg Psychiatry 1997 62 10 15 9010393 10.1136/jnnp.62.1.10 PMC486688

[B18] Li SC Schoenberg BS Wang CC Cheng XM Rui DY Bolis CL Schoenberg DG A prevalence survey of Parkinson's disease and other movement disorders in the People's Republic of China Arch Neurol 1985 42 655 657 4015461 10.1001/archneur.1985.04060070045013

[B19] Schrag A Ben-Shlomo Y Quinn NP Cross sectional prevalence survey of idiopathic Parkinson's disease and Parkinsonism in London BMJ 2000 321 21 22 10875828 10.1136/bmj.321.7252.21 PMC27420

[B20] de Lau LM Giesbergen PC de Rijk MC Hofman A Koudstaal PJ Breteler MM Incidence of parkinsonism and Parkinson disease in a general population: the Rotterdam Study Neurology 2004 63 1240 1244 15477545 10.1212/01.wnl.0000140706.52798.be

[B21] Van Den Eeden SK Tanner CM Bernstein AL Fross RD Leimpeter A Bloch DA Nelson LM Incidence of Parkinson's disease: variation by age, gender, and race/ethnicity Am J Epidemiol 2003 157 1015 1022 12777365 10.1093/aje/kwg068

[B22] Quinn N Critchley P Marsden CD Young onset Parkinson's disease Mov Disord 1987 2 73 91 3504266 10.1002/mds.870020201

[B23] Morris ME Movement disorders in people with Parkinson disease: a model for physical therapy Phys Ther 2000 80 578 597 10842411

[B24] Hughes AJ Daniel SE Kilford L Lees AJ Accuracy of clinical diagnosis of idiopathic Parkinson's disease: a clinico-pathological study of 100 cases J Neurol Neurosurg Psychiatry 1992 55 181 184 1564476 10.1136/jnnp.55.3.181 PMC1014720

[B25] Diamond SG Markham CH Hoehn MM McDowell FH Muenter MD Effect of age at onset on progression and mortality in Parkinson's disease Neurology 1989 39 1187 1190 2771070 10.1212/wnl.39.9.1187

[B26] Harada H Nishikawa S Takahashi K Epidemiology of Parkinson's disease in a Japanese city Arch Neurol 1983 40 151 154 6830454 10.1001/archneur.1983.04050030045008

[B27] Barbeau A Pourcher E New data on the genetics of Parkinson's disease Can J Neurol Sci 1982 9 53 60 7093827 10.1017/s031716710004364x

[B28] Schrag A Ben-Shlomo Y Brown R Marsden CD Quinn N Young-onset Parkinson's disease revisited--clinical features, natural history, and mortality Mov Disord 1998 13 885 894 9827611 10.1002/mds.870130605

[B29] Parkinson Study Group A controlled trial of rasagiline in early Parkinson disease: the TEMPO Study Arch Neurol 2002 59 1937 1943 12470183 10.1001/archneur.59.12.1937

[B30] Kurlan R Rubin AJ Miller C Rivera-Calimlim L Clarke A Shoulson I Duodenal delivery of levodopa for on-off fluctuations in parkinsonism: preliminary observations Ann Neurol 1986 20 262 265 3752968 10.1002/ana.410200213

[B31] Gibb WR Lees AJ A comparison of clinical and pathological features of young- and old-onset Parkinson's disease Neurology 1988 38 1402 1406 3412587 10.1212/wnl.38.9.1402

[B32] Pederzoli M Girotti F Scigliano G Aiello G Carella F Caraceni T L-DOPA long-term treatment in Parkinson's disease: age-related side effects Neurology 1983 33 1518 1522 6415513 10.1212/wnl.33.11.1518

[B33] Ishihara LS Cheesbrough A Brayne C Schrag A Estimated life expectancy of Parkinson's patients compared with the UK population J Neurol Neurosurg Psychiatry 2007 78 1304 1309 17400591 10.1136/jnnp.2006.100107 PMC2095626

[B34] Schrag A Hovris A Morley D Quinn N Jahanshahi M Young- versus older-onset Parkinson's disease: impact of disease and psychosocial consequences Mov Disord 2003 18 1250 1256 14639664 10.1002/mds.10527

[B35] Kostic V Przedborski S Flaster E Sternic N Early development of levodopa-induced dyskinesias and response fluctuations in young-onset Parkinson's disease Neurology 1991 41 202 205 1992362 10.1212/wnl.41.2_part_1.202

[B36] Albin RL Young AB Penney JB The functional anatomy of basal ganglia disorders Trends Neurosci 1989 12 366 375 2479133 10.1016/0166-2236(89)90074-x

[B37] Alexander GE DeLong MR Strick PL Parallel organization of functionally segregated circuits linking basal ganglia and cortex Annu Rev Neurosci 1986 9 357 381 3085570 10.1146/annurev.ne.09.030186.002041

[B38] Crossman AR Primate models of dyskinesia: the experimental approach to the study of basal ganglia-related involuntary movement disorders Neuroscience 1987 21 1 40 2955248 10.1016/0306-4522(87)90322-8

[B39] Alexander GE Crutcher MD Functional architecture of basal ganglia circuits: neural substrates of parallel processing Trends Neurosci 1990 13 266 271 1695401 10.1016/0166-2236(90)90107-l

[B40] Kravitz AV Freeze BS Parker PR Kay K Thwin MT Deisseroth K Kreitzer AC Regulation of parkinsonian motor behaviours by optogenetic control of basal ganglia circuitry Nature 2010 466 622 626 20613723 10.1038/nature09159 PMC3552484

[B41] Fox SH Katzenschlager R Lim SY Ravina B Seppi K Coelho M Poewe W Rascol O Goetz CG Sampaio C The Movement Disorder Society Evidence-Based Medicine Review Update: treatments for the motor symptoms of Parkinson's disease Mov Disord 2011 26 Suppl 3 S2 41 22021173 10.1002/mds.23829

[B42] Cenci MA Ohlin KE Odin P Current options and future possibilities for the treatment of dyskinesia and motor fluctuations in Parkinson's disease CNS Neurol Disord Drug Targets 2011 10 670 684 21838677 10.2174/187152711797247885

[B43] Birkmayer W Hornykiewicz O [The L-3,4-dioxyphenylalanine (DOPA)-effect in Parkinson-akinesia] Wien Klin Wochenschr 1961 73 787 788 13869404

[B44] Fahn S The history of dopamine and levodopa in the treatment of Parkinson's disease Mov Disord 2008 23 Suppl 3 S497 508 18781671 10.1002/mds.22028

[B45] Barbeau A Murphy GF Sourkes TL Excretion of dopamine in diseases of basal ganglia Science 1961 133 1706 1707 13686753 10.1126/science.133.3465.1706-a

[B46] Stern MB Marek KL Friedman J Hauser RA LeWitt PA Tarsy D Olanow CW Double-blind, randomized, controlled trial of rasagiline as monotherapy in early Parkinson's disease patients Mov Disord 2004 19 916 923 15300656 10.1002/mds.20145

[B47] Nutt JG Fellman JH Pharmacokinetics of levodopa Clin Neuropharmacol 1984 7 35 49 6367973 10.1097/00002826-198403000-00002

[B48] Bianchine JR Messiha FS Hsu TH Peripheral aromatic L-amino acids decarboxylase inhibitor in parkinsonism. II. Effect on metabolism of L-2- 14 C-dopa Clin Pharmacol Ther 1972 13 584 594 5042372 10.1002/cpt1972134584

[B49] Kuruma I Bartholini G Tissot R Fletscher A Comparative investigation of inhibitors of extracerebral dopa decarboxylase in man and rats J Pharm Pharmacol 1972 24 289 294 4402836 10.1111/j.2042-7158.1972.tb08988.x

[B50] Contin M Riva R Albani F Baruzzi A Pharmacokinetic optimisation in the treatment of Parkinson's disease Clin Pharmacokinet 1996 30 463 481 8792058 10.2165/00003088-199630060-00004

[B51] Nyholm D Pharmacokinetic optimisation in the treatment of Parkinson's disease: an update Clin Pharmacokinet 2006 45 109 136 16485914 10.2165/00003088-200645020-00001

[B52] Riederer P Youdim MB Monoamine oxidase activity and monoamine metabolism in brains of parkinsonian patients treated with l-deprenyl J Neurochem 1986 46 1359 1365 2420928 10.1111/j.1471-4159.1986.tb01747.x

[B53] Effect of deprenyl on the progression of disability in early Parkinson's disease. The Parkinson Study Group N Engl J Med 1989 321 1364 1371 2509910 10.1056/NEJM198911163212004

[B54] Palhagen S Heinonen EH Hagglund J Kaugesaar T Kontants H Maki-Ikola O Palm R Turunen J Selegiline delays the onset of disability in de novo parkinsonian patients. Swedish Parkinson Study Group Neurology 1998 51 520 525 9710028 10.1212/wnl.51.2.520

[B55] Seppi K Weintraub D Coelho M Perez-Lloret S Fox SH Katzenschlager R Hametner EM Poewe W Rascol O Goetz CG Sampaio C The Movement Disorder Society Evidence-Based Medicine Review Update: treatments for the non-motor symptoms of Parkinson's disease Mov Disord 2011 26 Suppl 3 S42 80 22021174 10.1002/mds.23884 PMC4020145

[B56] Watts RL The role of dopamine agonists in early Parkinson's disease Neurology 1997 49 Suppl 1 S34 48 9222273 10.1212/wnl.49.1_suppl_1.s34

[B57] Olanow CW The role of dopamine agonists in the treatment of early Parkinson's disease Neurology 2002 58 Suppl 1 S33 41 11909983 10.1212/wnl.58.suppl_1.s33

[B58] Scott RM Brody JA Cooper IS The effect of thalamotomy on the progress of unilateral Parkinson's disease J Neurosurg 1970 32 286 288 5416911 10.3171/jns.1970.32.3.0286

[B59] Duval C Panisset M Bertrand G Sadikot AF Evidence that ventrolateral thalamotomy may eliminate the supraspinal component of both pathological and physiological tremors Exp Brain Res 2000 132 216 222 10853946 10.1007/s002210000358

[B60] Duval C Panisset M Strafella AP Sadikot AF The impact of ventrolateral thalamotomy on tremor and voluntary motor behavior in patients with Parkinson's disease Exp Brain Res 2006 170 160 171 16328283 10.1007/s00221-005-0198-4

[B61] Duval C Strafella AP Sadikot AF The impact of ventrolateral thalamotomy on high-frequency components of tremor Clin Neurophysiol 2005 116 1391 1399 15978501 10.1016/j.clinph.2005.01.012

[B62] Atkinson JD Collins DL Bertrand G Peters TM Pike GB Sadikot AF Optimal location of thalamotomy lesions for tremor associated with Parkinson disease: a probabilistic analysis based on postoperative magnetic resonance imaging and an integrated digital atlas J Neurosurg 2002 96 854 866 12005392 10.3171/jns.2002.96.5.0854

[B63] Ohye C Higuchi Y Shibazaki T Hashimoto T Koyama T Hirai T Matsuda S Serizawa T Hori T Hayashi M Ochiai T Samura H Yamashiro K Gamma knife thalamotomy for Parkinson disease and essential tremor: a prospective multicenter study Neurosurgery 2012 70 526 535 discussion 535-526 21904267 10.1227/NEU.0b013e3182350893

[B64] Fox MW Ahlskog JE Kelly PJ Stereotactic ventrolateralis thalamotomy for medically refractory tremor in post-levodopa era Parkinson's disease patients J Neurosurg 1991 75 723 730 1919694 10.3171/jns.1991.75.5.0723

[B65] Hurtig HI Stern MB Thalamotomy for Parkinson's disease J Neurosurg 1985 62 163 165 3964852

[B66] Matsumoto K Shichijo F Fukami T Long-term follow-up review of cases of Parkinson's disease after unilateral or bilateral thalamotomy J Neurosurg 1984 60 1033 1044 6716138 10.3171/jns.1984.60.5.1033

[B67] Mosso JA Rand RW Management of parkinson's disease--combined therapy with levodopa and thalamotomy West J Med 1975 122 1 6 1109524 PMC1130254

[B68] Tasker RR Munz M Junn FS Kiss ZH Davis K Dostrovsky JO Lozano AM Deep brain stimulation and thalamotomy for tremor compared Acta Neurochir Suppl 1997 68 49 53 9233413 10.1007/978-3-7091-6513-3_9

[B69] Tasker RR Siqueira J Hawrylyshyn P Organ LW What happened to VIM thalamotomy for Parkinson's disease? Appl Neurophysiol 1983 46 68 83 6367656 10.1159/000101245

[B70] de Bie RM de Haan RJ Schuurman PR Esselink RA Bosch DA Speelman JD Morbidity and mortality following pallidotomy in Parkinson's disease: a systematic review Neurology 2002 58 1008 1012 11940683 10.1212/wnl.58.7.1008

[B71] De Bie RM Schuurman PR Esselink RA Bosch DA Speelman JD Bilateral pallidotomy in Parkinson's disease: a retrospective study Mov Disord 2002 17 533 538 12112203 10.1002/mds.10090

[B72] Esselink RA de Bie RM de Haan RJ Lenders MW Nijssen PC Staal MJ Smeding HM Schuurman PR Bosch DA Speelman JD Unilateral pallidotomy versus bilateral subthalamic nucleus stimulation in PD: a randomized trial Neurology 2004 62 201 207 14745054 10.1212/01.wnl.0000103235.12621.c3

[B73] Esselink RA de Bie RM de Haan RJ Lenders MW Nijssen PC van Laar T Schuurman PR Bosch DA Speelman JD Long-term superiority of subthalamic nucleus stimulation over pallidotomy in Parkinson disease Neurology 2009 73 151 153 19597136 10.1212/WNL.0b013e3181ad536c

[B74] Esselink RA de Bie RM de Haan RJ Steur EN Beute GN Portman AT Schuurman PR Bosch DA Speelman JD Unilateral pallidotomy versus bilateral subthalamic nucleus stimulation in Parkinson's disease: one year follow-up of a randomised observer-blind multi centre trial Acta Neurochir (Wien) 2006 148 1247 1255 discussion 1255 17072792 10.1007/s00701-006-0907-1

[B75] Smeding HM Esselink RA Schmand B Koning-Haanstra M Nijhuis I Wijnalda EM Speelman JD Unilateral pallidotomy versus bilateral subthalamic nucleus stimulation in PD--a comparison of neuropsychological effects J Neurol 2005 252 176 182 15729523 10.1007/s00415-005-0628-z

[B76] Coban A Hanagasi HA Karamursel S Barlas O Comparison of unilateral pallidotomy and subthalamotomy findings in advanced idiopathic Parkinson's disease Br J Neurosurg 2009 23 23 29 19234905 10.1080/02688690802507775

[B77] Bronstein JM DeSalles A DeLong MR Stereotactic pallidotomy in the treatment of Parkinson disease: an expert opinion Arch Neurol 1999 56 1064 1069 10488806 10.1001/archneur.56.9.1064

[B78] Gironell A Kulisevsky J Rami L Fortuny N Garcia-Sanchez C Pascual-Sedano B Effects of pallidotomy and bilateral subthalamic stimulation on cognitive function in Parkinson disease. A controlled comparative study J Neurol 2003 250 917 923 12928909 10.1007/s00415-003-1109-x

[B79] Hariz MI Bergenheim AT A 10-year follow-up review of patients who underwent Leksell's posteroventral pallidotomy for Parkinson disease J Neurosurg 2001 94 552 558 11302652 10.3171/jns.2001.94.4.0552

[B80] Intemann PM Masterman D Subramanian I DeSalles A Behnke E Frysinger R Bronstein JM Staged bilateral pallidotomy for treatment of Parkinson disease J Neurosurg 2001 94 437 444 11235949 10.3171/jns.2001.94.3.0437

[B81] Alvarez L Macias R Pavon N Lopez G Rodriguez-Oroz MC Rodriguez R Alvarez M Pedroso I Teijeiro J Fernandez R Casabona E Salazar S Maragoto C Carballo M García I Guridi J Juncos JL DeLong MR Obeso JA Therapeutic efficacy of unilateral subthalamotomy in Parkinson's disease: results in 89 patients followed for up to 36 months J Neurol Neurosurg Psychiatry 2009 80 979 985 19204026 10.1136/jnnp.2008.154948

[B82] Merello M Tenca E Perez Lloret S Martin ME Bruno V Cavanagh S Antico J Cerquetti D Leiguarda R Prospective randomized 1-year follow-up comparison of bilateral subthalamotomy versus bilateral subthalamic stimulation and the combination of both in Parkinson's disease patients: a pilot study Br J Neurosurg 2008 22 415 422 18568731 10.1080/02688690801971667

[B83] Alvarez L Macias R Lopez G Alvarez E Pavon N Rodriguez-Oroz MC Juncos JL Maragoto C Guridi J Litvan I Tolosa ES Koller W Vitek J DeLong MR Obeso JA Bilateral subthalamotomy in Parkinson's disease: initial and long-term response Brain 2005 128 570 583 15689366 10.1093/brain/awh397

[B84] Gill SS Heywood P Bilateral dorsolateral subthalamotomy for advanced Parkinson's disease Lancet 1997 350 1224 9652569 10.1016/s0140-6736(05)63455-1

[B85] Obeso JA Jahanshahi M Alvarez L Macias R Pedroso I Wilkinson L Pavon N Day B Pinto S Rodriguez-Oroz MC Tejeiro J Artieda J Talelli P Swayne O Rodríguez R Bhatia K Rodriguez-Diaz M Lopez G Guridi J Rothwell JC What can man do without basal ganglia motor output? The effect of combined unilateral subthalamotomy and pallidotomy in a patient with Parkinson's disease Exp Neurol 2009 220 283 292 19744484 10.1016/j.expneurol.2009.08.030

[B86] Patel NK Heywood P O'Sullivan K McCarter R Love S Gill SS Unilateral subthalamotomy in the treatment of Parkinson's disease Brain 2003 126 1136 1145 12690053 10.1093/brain/awg111

[B87] Su PC Tseng HM Subthalamotomy for end-stage severe Parkinson's disease Mov Disord 2002 17 625 627 author reply 627 12112226 10.1002/mds.10130

[B88] Su PC Tseng HM Liu HM Yen RF Liou HH Subthalamotomy for advanced Parkinson disease J Neurosurg 2002 97 598 606 12296644 10.3171/jns.2002.97.3.0598

[B89] Su PC Tseng HM Liu HM Yen RF Liou HH Treatment of advanced Parkinson's disease by subthalamotomy: one-year results Mov Disord 2003 18 531 538 12722167 10.1002/mds.10393

[B90] Tseng HM Su PC Liu HM Liou HH Yen RF Bilateral subthalamotomy for advanced Parkinson disease Surg Neurol 2007 68 Suppl 1 S43 50 discussion S50-41 17963922 10.1016/j.surneu.2007.05.058

[B91] Ponce FA Lozano AM Deep brain stimulation state of the art and novel stimulation targets Prog Brain Res 2010 184 311 324 20887882 10.1016/S0079-6123(10)84016-6

[B92] Videnovic A Metman LV Deep brain stimulation for Parkinson's disease: prevalence of adverse events and need for standardized reporting Mov Disord 2008 23 343 349 17987644 10.1002/mds.21753

[B93] Seijo FJ Alvarez-Vega MA Gutierrez JC Fdez-Glez F Lozano B Complications in subthalamic nucleus stimulation surgery for treatment of Parkinson's disease. Review of 272 procedures Acta Neurochir (Wien) 2007 149 867 875 discussion 876 17690838 10.1007/s00701-007-1267-1

[B94] Hu X Jiang X Zhou X Liang J Wang L Cao Y Liu J Jin A Yang P Avoidance and management of surgical and hardware-related complications of deep brain stimulation Stereotact Funct Neurosurg 2010 88 296 303 20588081 10.1159/000316762

[B95] Bronstein JM Tagliati M Alterman RL Lozano AM Volkmann J Stefani A Horak FB Okun MS Foote KD Krack P Pahwa R Henderson JM Hariz MI Bakay RA Rezai A Marks WJ Jr Moro E Vitek JL Weaver FM Gross RE DeLong MR Deep brain stimulation for Parkinson disease: an expert consensus and review of key issues Arch Neurol 2011 68 165 20937936 10.1001/archneurol.2010.260 PMC4523130

[B96] Lanotte M Verna G Panciani PP Taveggia A Zibetti M Lopiano L Ducati A Management of skin erosion following deep brain stimulation Neurosurg Rev 2009 32 111 114 discussion 114-115 18773232 10.1007/s10143-008-0158-0

[B97] Lyons KE Wilkinson SB Overman J Pahwa R Surgical and hardware complications of subthalamic stimulation: a series of 160 procedures Neurology 2004 63 612 616 15326230 10.1212/01.wnl.0000134650.91974.1a

[B98] Hariz MI Rehncrona S Quinn NP Speelman JD Wensing C Multicenter study on deep brain stimulation in Parkinson's disease: an independent assessment of reported adverse events at 4 years Mov Disord 2008 23 416 421 18067188 10.1002/mds.21888

[B99] Follett KA Torres-Russotto D Deep brain stimulation of globus pallidus interna, subthalamic nucleus, and pedunculopontine nucleus for Parkinson's disease: which target? Parkinsonism Relat Disord 2012 18 Suppl 1 S165 167 22166422 10.1016/S1353-8020(11)70051-7

[B100] Weaver FM Follett KA Stern M Luo P Harris CL Hur K Marks WJ Jr Rothlind J Sagher O Moy C Pahwa R Burchiel K Hogarth P Lai EC Duda JE Holloway K Samii A Horn S Bronstein JM Stoner G Starr PA Simpson R Baltuch G De Salles A Huang GD Reda DJ CSP 468 Study Group Randomized trial of deep brain stimulation for Parkinson disease: thirty-six-month outcomes Neurology 2012 79 55 65 22722632 10.1212/WNL.0b013e31825dcdc1 PMC3385495

[B101] Gervais-Bernard H Xie-Brustolin J Mertens P Polo G Klinger H Adamec D Broussolle E Thobois S Bilateral subthalamic nucleus stimulation in advanced Parkinson's disease: five year follow-up J Neurol 2009 256 225 233 19242649 10.1007/s00415-009-0076-2

[B102] Kleiner-Fisman G Herzog J Fisman DN Tamma F Lyons KE Pahwa R Lang AE Deuschl G Subthalamic nucleus deep brain stimulation: summary and meta-analysis of outcomes Mov Disord 2006 21 Suppl 14 S290 304 16892449 10.1002/mds.20962

[B103] Follett KA Weaver FM Stern M Hur K Harris CL Luo P Marks WJ Jr Rothlind J Sagher O Moy C Pahwa R Burchiel K Hogarth P Lai EC Duda JE Holloway K Samii A Horn S Bronstein JM Stoner G Starr PA Simpson R Baltuch G De Salles A Huang GD Reda DJ CSP 468 Study Group Pallidal versus subthalamic deep-brain stimulation for Parkinson's disease N Engl J Med 2010 362 2077 2091 20519680 10.1056/NEJMoa0907083

[B104] Odekerken VJ van Laar T Staal MJ Mosch A Hoffmann CF Nijssen PC Beute GN van Vugt JP Lenders MW Contarino MF Mink MS Bour LJ van den Munckhof P Schmand BA de Haan RJ Schuurman PR de Bie RM Subthalamic nucleus versus globus pallidus bilateral deep brain stimulation for advanced Parkinson's disease (NSTAPS study): a randomised controlled trial Lancet Neurol 2013 12 37 55 23168021 10.1016/S1474-4422(12)70264-8

[B105] Okun MS Foote KD Parkinson's disease DBS: what, when, who and why? The time has come to tailor DBS targets Expert Rev Neurother 2010 10 1847 1857 21384698 10.1586/ern.10.156 PMC3076937

[B106] Taba HA Wu SS Foote KD Hass CJ Fernandez HH Malaty IA Rodriguez RL Dai Y Zeilman PR Jacobson CE Okun MS A closer look at unilateral versus bilateral deep brain stimulation: results of the National Institutes of Health COMPARE cohort J Neurosurg 2010 113 1224 1229 20849215 10.3171/2010.8.JNS10312

[B107] Moro E Lozano AM Pollak P Agid Y Rehncrona S Volkmann J Kulisevsky J Obeso JA Albanese A Hariz MI Quinn NP Speelman JD Benabid AL Fraix V Mendes A Welter ML Houeto JL Cornu P Dormont D Tornqvist AL Ekberg R Schnitzler A Timmermann L Wojtecki L Gironell A Rodriguez-Oroz MC Guridi J Bentivoglio AR Contarino MF Romito L Long-term results of a multicenter study on subthalamic and pallidal stimulation in Parkinson's disease Mov Disord 2010 25 578 586 20213817 10.1002/mds.22735

[B108] Volkmann J Albanese A Kulisevsky J Tornqvist AL Houeto JL Pidoux B Bonnet AM Mendes A Benabid AL Fraix V Van Blercom N Xie J Obeso J Rodriguez-Oroz MC Guridi J Schnitzler A Timmermann L Gironell AA Molet J Pascual-Sedano B Rehncrona S Moro E Lang AC Lozano AM Bentivoglio AR Scerrati M Contarino MF Romito L Janssens M Agid Y Long-term effects of pallidal or subthalamic deep brain stimulation on quality of life in Parkinson's disease Mov Disord 2009 24 1154 1161 19412954 10.1002/mds.22496

[B109] Anderson VC Burchiel KJ Hogarth P Favre J Hammerstad JP Pallidal vs subthalamic nucleus deep brain stimulation in Parkinson disease Arch Neurol 2005 62 554 560 15824252 10.1001/archneur.62.4.554

[B110] Apetauerova D Ryan RK Ro SI Arle J Shils J Papavassiliou E Tarsy D End of day dyskinesia in advanced Parkinson's disease can be eliminated by bilateral subthalamic nucleus or globus pallidus deep brain stimulation Mov Disord 2006 21 1277 1279 16637040 10.1002/mds.20896

[B111] Romito LM Contarino MF Vanacore N Bentivoglio AR Scerrati M Albanese A Replacement of dopaminergic medication with subthalamic nucleus stimulation in Parkinson's disease: long-term observation Mov Disord 2009 24 557 563 19097175 10.1002/mds.22390

[B112] Benabid AL Torres N New targets for DBS Parkinsonism Relat Disord 2012 18 Suppl 1 S21 23 22166437 10.1016/S1353-8020(11)70009-8

[B113] Lee MS Rinne JO Marsden CD The pedunculopontine nucleus: its role in the genesis of movement disorders Yonsei Med J 2000 41 167 184 10817016 10.3349/ymj.2000.41.2.167

[B114] Moreau C Defebvre L Devos D Marchetti F Destee A Stefani A Peppe A STN versus PPN-DBS for alleviating freezing of gait: toward a frequency modulation approach? Mov Disord 2009 24 2164 2166 19705483 10.1002/mds.22743

[B115] Stefani A Lozano AM Peppe A Stanzione P Galati S Tropepi D Pierantozzi M Brusa L Scarnati E Mazzone P Bilateral deep brain stimulation of the pedunculopontine and subthalamic nuclei in severe Parkinson's disease Brain 2007 130 1596 1607 17251240 10.1093/brain/awl346

[B116] Yelnik J PPN or PPD, what is the target for deep brain stimulation in Parkinson's disease? Brain 2007 130 e79 author reply e80 17586558 10.1093/brain/awm138

[B117] Peppe A Gasbarra A Stefani A Chiavalon C Pierantozzi M Fermi E Stanzione P Caltagirone C Mazzone P Deep brain stimulation of CM/PF of thalamus could be the new elective target for tremor in advanced Parkinson's Disease? Parkinsonism Relat Disord 2008 14 501 504 18337153 10.1016/j.parkreldis.2007.11.005

[B118] Karlsson F Unger E Wahlgren S Blomstedt P Linder J Nordh E Zafar H van Doorn J Deep brain stimulation of caudal zona incerta and subthalamic nucleus in patients with Parkinson's disease: effects on diadochokinetic rate Parkinsons Dis 2011 2011 605607 doi: 10.4061/2011/605607 22007342 10.4061/2011/605607 PMC3191820

[B119] Lundgren S Saeys T Karlsson F Olofsson K Blomstedt P Linder J Nordh E Zafar H van Doorn J Deep brain stimulation of caudal zona incerta and subthalamic nucleus in patients with Parkinson's disease: effects on voice intensity Parkinsons Dis 2011 2011 658956 doi: 10.4061/2011/658956 22028987 10.4061/2011/658956 PMC3199057

[B120] Sundstedt S Olofsson K van Doorn J Linder J Nordh E Blomstedt P Swallowing function in Parkinson's patients following Zona Incerta deep brain stimulation Acta Neurol Scand 2012 126 350356 10.1111/j.1600-0404.2012.01658.x 22384826

[B121] Taira T Will ventralis intermedius deep brain stimulation for tremor be replaced by posterior subthalamic area or caudal zona incerta stimulation? World Neurosurg 2012 78 445 446 22381881 10.1016/j.wneu.2012.01.025

[B122] Hauser RA Schwarzschild MA Adenosine A2A receptor antagonists for Parkinson's disease: rationale, therapeutic potential and clinical experience Drugs Aging 2005 22 471 482 15974638 10.2165/00002512-200522060-00002

[B123] Hsieh PW Hung CF Fang JY Current prodrug design for drug discovery Curr Pharm Des 2009 15 2236 2250 19601825 10.2174/138161209788682523

[B124] Sozio P Cerasa LS Abbadessa A Di Stefano A Designing prodrugs for the treatment of Parkinson's disease Expert Opin Drug Discov 2012 7 385 406 22494466 10.1517/17460441.2012.677025

[B125] Politis M Wu K Loane C Kiferle L Molloy S Brooks DJ Piccini P Staging of serotonergic dysfunction in Parkinson's disease: an in vivo 11C-DASB PET study Neurobiol Dis 2010 40 216 221 20594979 10.1016/j.nbd.2010.05.028

[B126] Politis M Wu K Loane C Quinn NP Brooks DJ Rehncrona S Bjorklund A Lindvall O Piccini P Serotonergic neurons mediate dyskinesia side effects in Parkinson's patients with neural transplants Sci Transl Med 2010 2 38ra46 20592420 10.1126/scitranslmed.3000976

[B127] Dunnett SB Bjorklund A Lindvall O Cell therapy in Parkinson's disease - stop or go? Nat Rev Neurosci 2001 2 365 369 11331920 10.1038/35072572

[B128] Marks WJ Jr Bartus RT Siffert J Davis CS Lozano A Boulis N Vitek J Stacy M Turner D Verhagen L Bakay R Watts R Guthrie B Jankovic J Simpson R Tagliati M Alterman R Stern M Baltuch G Starr PA Larson PS Ostrem JL Nutt J Kieburtz K Kordower JH Olanow CW Gene delivery of AAV2-neurturin for Parkinson's disease: a double-blind, randomised, controlled trial Lancet Neurol 2010 9 1164 1172 20970382 10.1016/S1474-4422(10)70254-4

[B129] Marks WJ Jr Ostrem JL Verhagen L Starr PA Larson PS Bakay RA Taylor R Cahn-Weiner DA Stoessl AJ Olanow CW Bartus RT Safety and tolerability of intraputaminal delivery of CERE-120 (adeno-associated virus serotype 2-neurturin) to patients with idiopathic Parkinson's disease: an open-label, phase I trial Lancet Neurol 2008 7 400 408 18387850 10.1016/S1474-4422(08)70065-6

[B130] Gasmi M Herzog CD Brandon EP Cunningham JJ Ramirez GA Ketchum ET Bartus RT Striatal delivery of neurturin by CERE-120, an AAV2 vector for the treatment of dopaminergic neuron degeneration in Parkinson's disease Mol Ther 2007 15 62 68 17164776 10.1038/sj.mt.6300010

[B131] Herzog CD Dass B Holden JE Stansell J Gasmi M Tuszynski MH Bartus RT Kordower JH Striatal delivery of CERE-120, an AAV2 vector encoding human neurturin, enhances activity of the dopaminergic nigrostriatal system in aged monkeys Mov Disord 2007 22 1124 1132 17443702 10.1002/mds.21503

[B132] Kaplitt MG Feigin A Tang C Fitzsimons HL Mattis P Lawlor PA Bland RJ Young D Strybing K Eidelberg D During MJ Safety and tolerability of gene therapy with an adeno-associated virus (AAV) borne GAD gene for Parkinson's disease: an open label, phase I trial Lancet 2007 369 2097 2105 17586305 10.1016/S0140-6736(07)60982-9

[B133] LeWitt PA Rezai AR Leehey MA Ojemann SG Flaherty AW Eskandar EN Kostyk SK Thomas K Sarkar A Siddiqui MS Tatter SB Schwalb JM Poston KL Henderson JM Kurlan RM Richard IH Van Meter L Sapan CV During MJ Kaplitt MG Feigin A AAV2-GAD gene therapy for advanced Parkinson's disease: a double-blind, sham-surgery controlled, randomised trial Lancet Neurol 2011 10 309 319 21419704 10.1016/S1474-4422(11)70039-4

[B134] Luo J Kaplitt MG Fitzsimons HL Zuzga DS Liu Y Oshinsky ML During MJ Subthalamic GAD gene therapy in a Parkinson's disease rat model Science 2002 298 425 429 12376704 10.1126/science.1074549

[B135] Sgambato-Faure V Cenci MA Glutamatergic mechanisms in the dyskinesias induced by pharmacological dopamine replacement and deep brain stimulation for the treatment of Parkinson's disease Prog Neurobiol 2012 96 69 86 22075179 10.1016/j.pneurobio.2011.10.005

[B136] Krack P Batir A Van Blercom N Chabardes S Fraix V Ardouin C Koudsie A Limousin PD Benazzouz A LeBas JF Benabid AL Pollak P Five-year follow-up of bilateral stimulation of the subthalamic nucleus in advanced Parkinson's disease N Engl J Med 2003 349 1925 1934 14614167 10.1056/NEJMoa035275

[B137] Krack P Fraix V Mendes A Benabid AL Pollak P Postoperative management of subthalamic nucleus stimulation for Parkinson's disease Mov Disord 2002 17 Suppl 3 S188 197 11948776 10.1002/mds.10163

[B138] Limousin P Pollak P Hoffmann D Benazzouz A Perret JE Benabid AL Abnormal involuntary movements induced by subthalamic nucleus stimulation in parkinsonian patients Mov Disord 1996 11 231 235 8723137 10.1002/mds.870110303

[B139] Hagell P Piccini P Bjorklund A Brundin P Rehncrona S Widner H Crabb L Pavese N Oertel WH Quinn N Brooks DJ Lindvall O Dyskinesias following neural transplantation in Parkinson's disease Nat Neurosci 2002 5 627 628 12042822 10.1038/nn863

[B140] Freed CR Greene PE Breeze RE Tsai WY DuMouchel W Kao R Dillon S Winfield H Culver S Trojanowski JQ Eidelberg D Fahn S Transplantation of embryonic dopamine neurons for severe Parkinson's disease N Engl J Med 2001 344 710 719 11236774 10.1056/NEJM200103083441002

[B141] Politis M Dyskinesias after neural transplantation in Parkinson's disease: what do we know and what is next? BMC Med 2010 8 80 21126348 10.1186/1741-7015-8-80 PMC3003184

[B142] Ahlskog JE Muenter MD Frequency of levodopa-related dyskinesias and motor fluctuations as estimated from the cumulative literature Mov Disord 2001 16 448 458 11391738 10.1002/mds.1090

[B143] Fahn S The spectrum of levodopa-induced dyskinesias Ann Neurol 2000 47 Suppl 1 S2 9 discussion S9-11 10762127

[B144] Klawans HL Goetz C Bergen D Levodopa-induced myoclonus Arch Neurol 1975 32 330 334 1079721 10.1001/archneur.1975.00490470075011

[B145] Nutt JG Motor fluctuations and dyskinesia in Parkinson's disease Parkinsonism Relat Disord 2001 8 101 108 11489675 10.1016/s1353-8020(01)00024-4

[B146] Gour J Edwards R Lemieux S Ghassemi M Jog M Duval C Movement patterns of peak-dose levodopa-induced dyskinesias in patients with Parkinson's disease Brain Res Bull 2007 74 66 74 17683791 10.1016/j.brainresbull.2007.05.005

[B147] Fenney A Jog MS Duval C Short-term variability in amplitude and motor topography of whole-body involuntary movements in Parkinson's disease dyskinesias and in Huntington's chorea Clin Neurol Neurosurg 2008 110 160 167 18063471 10.1016/j.clineuro.2007.10.010

[B148] Chelaru MI Duval C Jog M Levodopa-induced dyskinesias detection based on the complexity of involuntary movements J Neurosci Methods 2010 186 81 89 19878692 10.1016/j.jneumeth.2009.10.015

[B149] Mann RK Edwards R Zhou J Jog M Duval C Intra- and inter-limb coherency during stance in non-dyskinetic and dyskinetic patients with Parkinson's disease Clin Neurol Neurosurg 2010 112 392 399 20206438 10.1016/j.clineuro.2010.02.003

[B150] Mann RK Edwards R Zhou J Fenney A Jog M Duval C Comparing movement patterns associated with Huntington's chorea and Parkinson's dyskinesia Exp Brain Res 2012 218 639 654 22434341 10.1007/s00221-012-3057-0

[B151] Hallett PJ Dunah AW Ravenscroft P Zhou S Bezard E Crossman AR Brotchie JM Standaert DG Alterations of striatal NMDA receptor subunits associated with the development of dyskinesia in the MPTP-lesioned primate model of Parkinson's disease Neuropharmacology 2005 48 503 516 15755478 10.1016/j.neuropharm.2004.11.008

[B152] Silverdale MA Kobylecki C Hallett PJ Li Q Dunah AW Ravenscroft P Bezard E Brotchie JM Synaptic recruitment of AMPA glutamate receptor subunits in levodopa-induced dyskinesia in the MPTP-lesioned nonhuman primate Synapse 2010 64 177 180 19852073 10.1002/syn.20739

[B153] Troiano AR de la Fuente-Fernandez R Sossi V Schulzer M Mak E Ruth TJ Stoessl AJ PET demonstrates reduced dopamine transporter expression in PD with dyskinesias Neurology 2009 72 1211 1216 19020294 10.1212/01.wnl.0000338631.73211.56

[B154] Schrag A Quinn N Dyskinesias and motor fluctuations in Parkinson's disease. A community-based study Brain 2000 123 2297 2305 11050029 10.1093/brain/123.11.2297

[B155] Sharma JC Macnamara L Hasoon M Vassallo M Ross I Cascade of levodopa dose and weight-related dyskinesia in Parkinson's disease (LD-WD-PD cascade) Parkinsonism Relat Disord 2006 12 499 505 16935018 10.1016/j.parkreldis.2006.07.002

[B156] Bachmann CG Trenkwalder C Body weight in patients with Parkinson's disease Mov Disord 2006 21 1824 1830 16958133 10.1002/mds.21068

[B157] Guigoni C Bezard E Involvement of canonical and non-canonical D1 dopamine receptor signalling pathways in L-DOPA-induced dyskinesia Parkinsonism Relat Disord 2009 15 Suppl 3 S64 67 20083011 10.1016/S1353-8020(09)70783-7

[B158] Berthet A Porras G Doudnikoff E Stark H Cador M Bezard E Bloch B Pharmacological analysis demonstrates dramatic alteration of D1 dopamine receptor neuronal distribution in the rat analog of L-DOPA-induced dyskinesia J Neurosci 2009 29 4829 4835 19369551 10.1523/JNEUROSCI.5884-08.2009 PMC6665326

[B159] Fahn S A new look at levodopa based on the ELLDOPA study J Neural Transm Suppl 2006 419 426 17017562 10.1007/978-3-211-45295-0_63

[B160] Fahn S Parkinson disease, the effect of levodopa, and the ELLDOPA trial. Earlier vs Later L-DOPA Arch Neurol 1999 56 529 535 10328247 10.1001/archneur.56.5.529

[B161] Aubert I Guigoni C Hakansson K Li Q Dovero S Barthe N Bioulac BH Gross CE Fisone G Bloch B Bezard E Increased D1 dopamine receptor signaling in levodopa-induced dyskinesia Ann Neurol 2005 57 17 26 15514976 10.1002/ana.20296

[B162] Cenci MA Dopamine dysregulation of movement control in L-DOPA-induced dyskinesia Trends Neurosci 2007 30 236 243 17400300 10.1016/j.tins.2007.03.005

[B163] Bedard PJ Mancilla BG Blanchette P Gagnon C Di Paolo T Levodopa-induced dyskinesia: facts and fancy. What does the MPTP monkey model tell us? Can J Neurol Sci 1992 19 1 Suppl 134 137 1571858

[B164] Carta AR Tronci E Pinna A Morelli M Different responsiveness of striatonigral and striatopallidal neurons to L-DOPA after a subchronic intermittent L-DOPA treatment Eur J Neurosci 2005 21 1196 1204 15813929 10.1111/j.1460-9568.2005.03944.x

[B165] Venton BJ Zhang H Garris PA Phillips PE Sulzer D Wightman RM Real-time decoding of dopamine concentration changes in the caudate-putamen during tonic and phasic firing J Neurochem 2003 87 1284 1295 14622108 10.1046/j.1471-4159.2003.02109.x

[B166] Tedroff J Pedersen M Aquilonius SM Hartvig P Jacobsson G Langstrom B Levodopa-induced changes in synaptic dopamine in patients with Parkinson's disease as measured by [11C]raclopride displacement and PET Neurology 1996 46 1430 1436 8628494 10.1212/wnl.46.5.1430

[B167] de la Fuente-Fernandez R Sossi V Huang Z Furtado S Lu JQ Calne DB Ruth TJ Stoessl AJ Levodopa-induced changes in synaptic dopamine levels increase with progression of Parkinson's disease: implications for dyskinesias Brain 2004 127 2747 2754 15329355 10.1093/brain/awh290

[B168] Goulet M Morissette M Calon F Blanchet PJ Falardeau P Bedard PJ Di Paolo T Continuous or pulsatile chronic D2 dopamine receptor agonist (U91356A) treatment of drug-naive 4-phenyl-1,2,3,6-tetrahydropyridine monkeys differentially regulates brain D1 and D2 receptor expression: in situ hybridization histochemical analysis Neuroscience 1997 79 497 507 9200732 10.1016/s0306-4522(96)00689-6

[B169] Morissette M Goulet M Soghomonian JJ Blanchet PJ Calon F Bedard PJ Di Paolo T Preproenkephalin mRNA expression in the caudate-putamen of MPTP monkeys after chronic treatment with the D2 agonist U91356A in continuous or intermittent mode of administration: comparison with L-DOPA therapy Brain Res Mol Brain Res 1997 49 55 62 9387863 10.1016/s0169-328x(97)00123-x

[B170] Lavoie B Parent A Immunohistochemical study of the serotoninergic innervation of the basal ganglia in the squirrel monkey J Comp Neurol 1990 299 1 16 2212111 10.1002/cne.902990102

[B171] Arai R Karasawa N Geffard M Nagatsu I L-DOPA is converted to dopamine in serotonergic fibers of the striatum of the rat: a double-labeling immunofluorescence study Neurosci Lett 1995 195 195 198 8584208 10.1016/0304-3940(95)11817-g

[B172] Carta M Carlsson T Kirik D Bjorklund A Dopamine released from 5-HT terminals is the cause of L-DOPA-induced dyskinesia in parkinsonian rats Brain 2007 130 1819 1833 17452372 10.1093/brain/awm082

[B173] Rylander D Parent M O'Sullivan SS Dovero S Lees AJ Bezard E Descarries L Cenci MA Maladaptive plasticity of serotonin axon terminals in levodopa-induced dyskinesia Ann Neurol 2010 68 619 628 20882603 10.1002/ana.22097

[B174] Guigoni C Doudnikoff E Li Q Bloch B Bezard E Altered D(1) dopamine receptor trafficking in parkinsonian and dyskinetic non-human primates Neurobiol Dis 2007 26 452 463 17350277 10.1016/j.nbd.2007.02.001

[B175] Gerfen CR Miyachi S Paletzki R Brown P D1 dopamine receptor supersensitivity in the dopamine-depleted striatum results from a switch in the regulation of ERK1/2/MAP kinase J Neurosci 2002 22 5042 5054 12077200 10.1523/JNEUROSCI.22-12-05042.2002 PMC6757735

[B176] Oh JD Russell DS Vaughan CL Chase TN Enhanced tyrosine phosphorylation of striatal NMDA receptor subunits: effect of dopaminergic denervation and L-DOPA administration Brain Res 1998 813 150 159 9824689 10.1016/s0006-8993(98)01049-x

[B177] Chase TN Oh JD Striatal dopamine- and glutamate-mediated dysregulation in experimental parkinsonism Trends Neurosci 2000 23 10 Suppl S86 91 11052225 10.1016/s1471-1931(00)00018-5

[B178] Rylander D Iderberg H Li Q Dekundy A Zhang J Li H Baishen R Danysz W Bezard E Cenci MA A mGluR5 antagonist under clinical development improves L-DOPA-induced dyskinesia in parkinsonian rats and monkeys Neurobiol Dis 2010 39 352 361 20452425 10.1016/j.nbd.2010.05.001

[B179] Rylander D Recchia A Mela F Dekundy A Danysz W Cenci MA Pharmacological modulation of glutamate transmission in a rat model of L-DOPA-induced dyskinesia: effects on motor behavior and striatal nuclear signaling J Pharmacol Exp Ther 2009 330 227 235 19357321 10.1124/jpet.108.150425 PMC2700169

[B180] Mela F Marti M Dekundy A Danysz W Morari M Cenci MA Antagonism of metabotropic glutamate receptor type 5 attenuates L-DOPA-induced dyskinesia and its molecular and neurochemical correlates in a rat model of Parkinson's disease J Neurochem 2007 101 483 497 17359492 10.1111/j.1471-4159.2007.04456.x

[B181] Levandis G Bazzini E Armentero MT Nappi G Blandini F Systemic administration of an mGluR5 antagonist, but not unilateral subthalamic lesion, counteracts L-DOPA-induced dyskinesias in a rodent model of Parkinson's disease Neurobiol Dis 2008 29 161 168 17933546 10.1016/j.nbd.2007.08.011

[B182] Ohlin KE Francardo V Lindgren HS Sillivan SE O'Sullivan SS Luksik AS Vassoler FM Lees AJ Konradi C Cenci MA Vascular endothelial growth factor is upregulated by L-DOPA in the parkinsonian brain: implications for the development of dyskinesia Brain 2011 134 2339 2357 21771855 10.1093/brain/awr165 PMC3155708

[B183] Fredduzzi S Moratalla R Monopoli A Cuellar B Xu K Ongini E Impagnatiello F Schwarzschild MA Chen JF Persistent behavioral sensitization to chronic L-DOPA requires A2A adenosine receptors J Neurosci 2002 22 1054 1062 11826134 10.1523/JNEUROSCI.22-03-01054.2002 PMC6758487

[B184] Xiao D Bastia E Xu YH Benn CL Cha JH Peterson TS Chen JF Schwarzschild MA Forebrain adenosine A2A receptors contribute to L-3,4-dihydroxyphenylalanine-induced dyskinesia in hemiparkinsonian mice J Neurosci 2006 26 13548 13555 17192438 10.1523/JNEUROSCI.3554-06.2006 PMC6674727

[B185] Xiao D Cassin JJ Healy B Burdett TC Chen JF Fredholm BB Schwarzschild MA Deletion of adenosine A(1) or A((2)A) receptors reduces L-3,4-dihydroxyphenylalanine-induced dyskinesia in a model of Parkinson's disease Brain Res 2011 1367 310 318 20828543 10.1016/j.brainres.2010.08.099 PMC3005012

[B186] Bibbiani F Oh JD Petzer JP Castagnoli N Jr Chen JF Schwarzschild MA Chase TN A2A antagonist prevents dopamine agonist-induced motor complications in animal models of Parkinson's disease Exp Neurol 2003 184 285 294 14637099 10.1016/s0014-4886(03)00250-4

[B187] Zeng BY Pearce RK MacKenzie GM Jenner P Alterations in preproenkephalin and adenosine-2a receptor mRNA, but not preprotachykinin mRNA correlate with occurrence of dyskinesia in normal monkeys chronically treated with L-DOPA Eur J Neurosci 2000 12 1096 1104 10762340 10.1046/j.1460-9568.2000.00988.x

[B188] Calon F Dridi M Hornykiewicz O Bedard PJ Rajput AH Di Paolo T Increased adenosine A2A receptors in the brain of Parkinson's disease patients with dyskinesias Brain 2004 127 1075 1084 15033896 10.1093/brain/awh128

[B189] Ochi M Shiozaki S Kase H Adenosine A(2A) receptor-mediated modulation of GABA and glutamate release in the output regions of the basal ganglia in a rodent model of Parkinson's disease Neuroscience 2004 127 223 231 15219684 10.1016/j.neuroscience.2004.04.050

[B190] Mela F Marti M Bido S Cenci MA Morari M In vivo evidence for a differential contribution of striatal and nigral D1 and D2 receptors to L-DOPA induced dyskinesia and the accompanying surge of nigral amino acid levels Neurobiol Dis 2012 45 573 582 22001605 10.1016/j.nbd.2011.09.015

[B191] Robelet S Melon C Guillet B Salin P Kerkerian-Le Goff L Chronic L-DOPA treatment increases extracellular glutamate levels and GLT1 expression in the basal ganglia in a rat model of Parkinson's disease Eur J Neurosci 2004 20 1255 1266 15341597 10.1111/j.1460-9568.2004.03591.x

[B192] Boulet S Lacombe E Carcenac C Feuerstein C Sgambato-Faure V Poupard A Savasta M Subthalamic stimulation-induced forelimb dyskinesias are linked to an increase in glutamate levels in the substantia nigra pars reticulata J Neurosci 2006 26 10768 10776 17050715 10.1523/JNEUROSCI.3065-06.2006 PMC6674740

[B193] Dupre KB Ostock CY Eskow Jaunarajs KL Button T Savage LM Wolf W Bishop C Local modulation of striatal glutamate efflux by serotonin 1A receptor stimulation in dyskinetic, hemiparkinsonian rats Exp Neurol 2011 229 288 299 21352823 10.1016/j.expneurol.2011.02.012 PMC3100430

[B194] Konradi C Westin JE Carta M Eaton ME Kuter K Dekundy A Lundblad M Cenci MA Transcriptome analysis in a rat model of L-DOPA-induced dyskinesia Neurobiol Dis 2004 17 219 236 15474360 10.1016/j.nbd.2004.07.005 PMC4208672

[B195] Rico AJ Barroso-Chinea P Conte-Perales L Roda E Gomez-Bautista V Gendive M Obeso JA Lanciego JL A direct projection from the subthalamic nucleus to the ventral thalamus in monkeys Neurobiol Dis 2010 39 381 392 20452426 10.1016/j.nbd.2010.05.004

[B196] Lieu CA Subramanian T The interhemispheric connections of the striatum: Implications for Parkinson's disease and drug-induced dyskinesias Brain Res Bull 2012 87 1 9 21963946 10.1016/j.brainresbull.2011.09.013 PMC3246032

[B197] Lieu CA Deogaonkar M Bakay RA Subramanian T Dyskinesias do not develop after chronic intermittent levodopa therapy in clinically hemiparkinsonian rhesus monkeys Parkinsonism Relat Disord 2011 17 34 39 21074478 10.1016/j.parkreldis.2010.10.010 PMC3053121

[B198] Van Gerpen JA Kumar N Bower JH Weigand S Ahlskog JE Levodopa-associated dyskinesia risk among Parkinson disease patients in Olmsted County, Minnesota, 1976-1990 Arch Neurol 2006 63 205 209 16476808 10.1001/archneur.63.2.205

[B199] Biglan K Holloway RG Initial treatment of early Parkinson's disease: a review of recent, randomized controlled trials Curr Neurol Neurosci Rep 2001 1 329 336 11898538 10.1007/s11910-001-0086-7

[B200] Olanow CW Rascol O Hauser R Feigin PD Jankovic J Lang A Langston W Melamed E Poewe W Stocchi F Tolosa E ADAGIO Study Investigators A double-blind, delayed-start trial of rasagiline in Parkinson's disease N Engl J Med 2009 361 1268 1278 19776408 10.1056/NEJMoa0809335

[B201] Samanta J Hauser RA Duodenal levodopa infusion for the treatment of Parkinson's disease Expert Opin Pharmacother 2007 8 657 664 17376020 10.1517/14656566.8.5.657

[B202] Cedarbaum JM The promise and limitations of controlled-release oral levodopa administration Clin Neuropharmacol 1989 12 147 166 2663145 10.1097/00002826-198906000-00001

[B203] Manson AJ Turner K Lees AJ Apomorphine monotherapy in the treatment of refractory motor complications of Parkinson's disease: long-term follow-up study of 64 patients Mov Disord 2002 17 1235 1241 12465062 10.1002/mds.10281

[B204] Nutt JG Obeso JA Stocchi F Continuous dopamine-receptor stimulation in advanced Parkinson's disease Trends Neurosci 2000 23 10 Suppl S109 115 11052228 10.1016/s1471-1931(00)00029-x

[B205] Olanow CW Obeso JA Stocchi F Continuous dopamine-receptor treatment of Parkinson's disease: scientific rationale and clinical implications Lancet Neurol 2006 5 677 687 16857573 10.1016/S1474-4422(06)70521-X

[B206] Nutt JG Continuous dopaminergic stimulation: Is it the answer to the motor complications of Levodopa? Mov Disord 2007 22 1 9 16958130 10.1002/mds.21060

[B207] Eggert K Schrader C Hahn M Stamelou M Russmann A Dengler R Oertel W Odin P Continuous jejunal levodopa infusion in patients with advanced parkinson disease: practical aspects and outcome of motor and non-motor complications Clin Neuropharmacol 2008 31 151 166 18520982 10.1097/wnf.0b013e31814b113e

[B208] Schmidt WJ Lebsanft H Heindl M Gerlach M Gruenblatt E Riederer P Mayerhofer A Scheller DK Continuous versus pulsatile administration of rotigotine in 6-OHDA-lesioned rats: contralateral rotations and abnormal involuntary movements J Neural Transm 2008 115 1385 1392 18726139 10.1007/s00702-008-0102-z

[B209] Jenner P McCreary AC Scheller DK Continuous drug delivery in early- and late-stage Parkinson's disease as a strategy for avoiding dyskinesia induction and expression J Neural Transm 2011 118 1691 1702 21881838 10.1007/s00702-011-0703-9

[B210] Katzenschlager R Head J Schrag A Ben-Shlomo Y Evans A Lees AJ Fourteen-year final report of the randomized PDRG-UK trial comparing three initial treatments in PD Neurology 2008 71 474 480 18579806 10.1212/01.wnl.0000310812.43352.66

[B211] Rascol O Brooks DJ Korczyn AD De Deyn PP Clarke CE Lang AE A five-year study of the incidence of dyskinesia in patients with early Parkinson's disease who were treated with ropinirole or levodopa. 056 Study Group N Engl J Med 2000 342 1484 1491 10816186 10.1056/NEJM200005183422004

[B212] Rinne UK Bracco F Chouza C Dupont E Gershanik O Marti Masso JF Montastruc JL Marsden CD Early treatment of Parkinson's disease with cabergoline delays the onset of motor complications. Results of a double-blind levodopa controlled trial. The PKDS009 Study Group Drugs 1998 55 Suppl 1 23 30 9483167 10.2165/00003495-199855001-00004

[B213] Oertel WH Wolters E Sampaio C Gimenez-Roldan S Bergamasco B Dujardin M Grosset DG Arnold G Leenders KL Hundemer HP Lledó A Wood A Frewer P Schwarz J Pergolide versus levodopa monotherapy in early Parkinson's disease patients: The PELMOPET study Mov Disord 2006 21 343 353 16211594 10.1002/mds.20724

[B214] Stowe RL Ives NJ Clarke C van Hilten J Ferreira J Hawker RJ Shah L Wheatley K Gray R Dopamine agonist therapy in early Parkinson's disease Cochrane Database Syst Rev 2008 2 CD006564 18425954 10.1002/14651858.CD006564.pub2 PMC12740267

[B215] Potenza MN Voon V Weintraub D Drug insight: impulse control disorders and dopamine therapies in Parkinson's disease Nat Clin Pract Neurol 2007 3 664 672 18046439 10.1038/ncpneuro0680

[B216] Metman LV Del Dotto P LePoole K Konitsiotis S Fang J Chase TN Amantadine for levodopa-induced dyskinesias: a 1-year follow-up study Arch Neurol 1999 56 1383 1386 10555659 10.1001/archneur.56.11.1383

[B217] Del Dotto P Pavese N Gambaccini G Bernardini S Metman LV Chase TN Bonuccelli U Intravenous amantadine improves levadopa-induced dyskinesias: an acute double-blind placebo-controlled study Mov Disord 2001 16 3 515 520 11391748 10.1002/mds.1112

[B218] Luginger E Wenning GK Bosch S Poewe W Beneficial effects of amantadine on L-DOPA-induced dyskinesias in Parkinson's disease Mov Disord 2000 15 873 878 11009193 10.1002/1531-8257(200009)15:5<873::aid-mds1017>3.0.co;2-i

[B219] Meltzer HY An overview of the mechanism of action of clozapine J Clin Psychiatry 1994 55 Suppl B 47 52 7961573

[B220] Meltzer HY Bastani B Ramirez L Matsubara S Clozapine: new research on efficacy and mechanism of action Eur Arch Psychiatry Neurol Sci 1989 238 332 339 2569975 10.1007/BF00449814

[B221] Miyamoto S Duncan GE Marx CE Lieberman JA Treatments for schizophrenia: a critical review of pharmacology and mechanisms of action of antipsychotic drugs Mol Psychiatry 2005 10 79 104 15289815 10.1038/sj.mp.4001556

[B222] Durif F Debilly B Galitzky M Morand D Viallet F Borg M Thobois S Broussolle E Rascol O Clozapine improves dyskinesias in Parkinson disease: a double-blind, placebo-controlled study Neurology 2004 62 381 388 14872017 10.1212/01.wnl.0000110317.52453.6c

[B223] Alvir JM Lieberman JA Safferman AZ Schwimmer JL Schaaf JA Clozapine-induced agranulocytosis. Incidence and risk factors in the United States N Engl J Med 1993 329 162 167 8515788 10.1056/NEJM199307153290303

[B224] Haas SJ Hill R Krum H Liew D Tonkin A Demos L Stephan K McNeil J Clozapine-associated myocarditis: a review of 116 cases of suspected myocarditis associated with the use of clozapine in Australia during 1993-2003 Drug Saf 2007 30 47 57 17194170 10.2165/00002018-200730010-00005

[B225] Evidente VG Premkumar AP Adler CH Caviness JN Driver-Dunckley E Lyons MK Medication dose reductions after pallidal versus subthalamic stimulation in patients with Parkinson's disease Acta Neurol Scand 2011 124 211 214 20969559 10.1111/j.1600-0404.2010.01455.x

[B226] Vitek JL Bakay RA Freeman A Evatt M Green J McDonald W Haber M Barnhart H Wahlay N Triche S Mewes K Chockkan V Zhang JY DeLong MR Randomized trial of pallidotomy versus medical therapy for Parkinson's disease Ann Neurol 2003 53 558 569 12730989 10.1002/ana.10517

[B227] Lewitt PA Hauser RA Lu M Nicholas AP Weiner W Coppard N Leinonen M Savola JM Randomized clinical trial of fipamezole for dyskinesia in Parkinson disease (FJORD study) Neurology 2012 79 163 169 22744665 10.1212/WNL.0b013e31825f0451

[B228] Savola JM Hill M Engstrom M Merivuori H Wurster S McGuire SG Fox SH Crossman AR Brotchie JM Fipamezole (JP-1730) is a potent alpha2 adrenergic receptor antagonist that reduces levodopa-induced dyskinesia in the MPTP-lesioned primate model of Parkinson's disease Mov Disord 2003 18 872 883 12889076 10.1002/mds.10464

[B229] Berg D Godau J Trenkwalder C Eggert K Csoti I Storch A Huber H Morelli-Canelo M Stamelou M Ries V Wolz M Schneider C Di Paolo T Gasparini F Hariry S Vandemeulebroecke M Abi-Saab W Cooke K Johns D Gomez-Mancilla B AFQ056 treatment of levodopa-induced dyskinesias: results of 2 randomized controlled trials Mov Disord 2011 26 1243 1250 21484867 10.1002/mds.23616

[B230] Lindsley CW Hopkins CR Metabotropic glutamate receptor 4 (mGlu4)-positive allosteric modulators for the treatment of Parkinson's disease: historical perspective and review of the patent literature Expert opinion on therapeutic patents 2012 22 5 461 481 22506633 10.1517/13543776.2012.679437

[B231] Chase TN Bibbiani F Bara-Jimenez W Dimitrova T Oh-Lee JD Translating A2A antagonist KW6002 from animal models to parkinsonian patients Neurology 2003 61 11 Suppl 6 S107 111 14663022 10.1212/01.wnl.0000095223.08711.48

[B232] Berg D Godau J Trenkwalder C Eggert K Csoti I Storch A Gasparini F Hariry S Vandemeulebroecke M Johns D AFQ056 treatment of severe levodopa induced dyskinesias: proof of concept study Mov Disord 2010 25 Suppl 2 S290

[B233] Baas H Dyskinesia in Parkinson's disease. Pathophysiology and clinical risk factors J Neurol 2000 247 Suppl 4 IV/12 16 10.1007/pl00007767 11199809

[B234] Grandas F Galiano ML Tabernero C Risk factors for levodopa-induced dyskinesias in Parkinson's disease J Neurol 1999 246 1127 1133 10653303 10.1007/s004150050530

[B235] Kurtzke JF Bennett DR Berg BO Beringer GB Goldstein M Vates TS Jr Neurologists in the United States--past, present, and future Neurology 1986 36 1576 1582 3785671 10.1212/wnl.36.12.1576

[B236] World Health Organization, World Federation of Neurology Atlas Country Resources for Neurological Disorders 2004 Geneva

[B237] Canadian Institute for Health Information Supply, Distribution and Migration of Canadian Physicians 2008 Health Human Resources. Ottawa 101

[B238] Dorsey ER Constantinescu R Thompson JP Biglan KM Holloway RG Kieburtz K Marshall FJ Ravina BM Schifitto G Siderowf A Tanner CM Projected number of people with Parkinson disease in the most populous nations, 2005 through 2030 Neurology 2007 68 384 386 17082464 10.1212/01.wnl.0000247740.47667.03

[B239] Guttman M Slaughter PM Theriault ME DeBoer DP Naylor CD Parkinsonism in Ontario: physician utilization Can J Neurol Sci 2002 29 221 226 12195610 10.1017/s0317167100001980

[B240] Duncan RP Earhart GM Measuring participation in individuals with Parkinson disease: relationships with disease severity, quality of life, and mobility Disability and rehabilitation 2011 33 1440 1446 21091047 10.3109/09638288.2010.533245

[B241] Greene SM Griffin WA Symptom study in context: effects of marital quality on signs of Parkinson's disease during patient-spouse interaction Psychiatry 1998 61 35 45 9595594 10.1080/00332747.1998.11024817

[B242] Garland B The psychosocial impact of late-stage Parkinson's disease J Neurosci Nurs 2004 36 184 15366541

[B243] Calne SM The psychosocial impact of late-stage Parkinson's disease J Neurosci Nurs 2003 35 306 313 14713096 10.1097/01376517-200312000-00004

[B244] Marras C Lang A Krahn M Tomlinson G Naglie G Quality of life in early Parkinson's disease: impact of dyskinesias and motor fluctuations Mov Disord 2004 19 22 28 14743356 10.1002/mds.10642

[B245] Zach M Friedman A Slawek J Derejko M Quality of life in Polish patients with long-lasting Parkinson's disease Mov Disord 2004 19 667 672 15197705 10.1002/mds.10698

[B246] Hely MA Morris JG Reid WG Trafficante R Sydney Multicenter Study of Parkinson's disease: non-L-DOPA-responsive problems dominate at 15 years Mov Disord 2005 20 190 199 15551331 10.1002/mds.20324

[B247] Hung SW Adeli GM Arenovich T Fox SH Lang AE Patient perception of dyskinesia in Parkinson's disease J Neurol Neurosurg Psychiatry 2010 81 1112 1115 20667858 10.1136/jnnp.2009.173286

[B248] Jenkinson PM Edelstyn NM Stephens R Ellis SJ Why are some Parkinson disease patients unaware of their dyskinesias? Cogn Behav Neurol 2009 22 117 121 19506428 10.1097/WNN.0b013e3181a722b0

[B249] Soh SE Morris ME McGinley JL Determinants of health-related quality of life in Parkinson's disease: a systematic review Parkinsonism Relat Disord 2011 17 1 9 20833572 10.1016/j.parkreldis.2010.08.012

[B250] Winter Y von Campenhausen S Arend M Longo K Boetzel K Eggert K Oertel WH Dodel R Barone P Health-related quality of life and its determinants in Parkinson's disease: results of an Italian cohort study Parkinsonism Relat Disord 2011 17 265 269 21310647 10.1016/j.parkreldis.2011.01.003

[B251] Rahman S Griffin HJ Quinn NP Jahanshahi M Quality of life in Parkinson's disease: the relative importance of the symptoms Mov Disord 2008 23 1428 1434 18543333 10.1002/mds.21667

[B252] Muller T Russ H Levodopa, motor fluctuations and dyskinesia in Parkinson's disease Expert Opin Pharmacother 2006 7 1715 1730 16925499 10.1517/14656566.7.13.1715

[B253] Montel S Bonnet AM Bungener C Quality of life in relation to mood, coping strategies, and dyskinesia in Parkinson's disease J Geriatr Psychiatry Neurol 2009 22 95 102 19150974 10.1177/0891988708328219

[B254] Damiano AM McGrath MM Willian MK Snyder CF LeWitt PA Reyes PF Richter RR Means ED Evaluation of a measurement strategy for Parkinson's disease: assessing patient health-related quality of life Qual Life Res 2000 9 87 100 10981209 10.1023/a:1008928321652

[B255] Chapuis S Ouchchane L Metz O Gerbaud L Durif F Impact of the motor complications of Parkinson's disease on the quality of life Mov Disord 2005 20 224 230 15384126 10.1002/mds.20279

[B256] Ashburn A Stack E Pickering RM Ward CD A community-dwelling sample of people with Parkinson's disease: characteristics of fallers and non-fallers Age Ageing 2001 30 47 52 11322672 10.1093/ageing/30.1.47

[B257] Stevenson JK Talebifard P Ty E Oishi MM McKeown MJ Dyskinetic Parkinson's disease patients demonstrate motor abnormalities off medication Exp Brain Res 2011 214 471 479 21877102 10.1007/s00221-011-2845-2

[B258] Marinus J Leentjens AF Visser M Stiggelbout AM van Hilten JJ Evaluation of the hospital anxiety and depression scale in patients with Parkinson's disease Clin Neuropharmacol 2002 25 318 324 12469006 10.1097/00002826-200211000-00008

[B259] Marinus J Visser M Martinez-Martin P van Hilten JJ Stiggelbout AM A short psychosocial questionnaire for patients with Parkinson's disease: the SCOPA-PS J Clin Epidemiol 2003 56 61 67 12589871 10.1016/s0895-4356(02)00569-3

[B260] Pechevis M Clarke CE Vieregge P Khoshnood B Deschaseaux-Voinet C Berdeaux G Ziegler M Effects of dyskinesias in Parkinson's disease on quality of life and health-related costs: a prospective European study Eur J Neurol 2005 12 956 963 16324089 10.1111/j.1468-1331.2005.01096.x

[B261] Dissanayaka NN Sellbach A Matheson S O'Sullivan JD Silburn PA Byrne GJ Marsh R Mellick GD Anxiety disorders in Parkinson's disease: prevalence and risk factors Mov Disord 2010 25 838 845 20461800 10.1002/mds.22833

[B262] Menza MA Sage J Marshall E Cody R Duvoisin R Mood changes and "on-off" phenomena in Parkinson's disease Mov Disord 1990 5 148 151 2325676 10.1002/mds.870050210

[B263] Henderson R Kurlan R Kersun JM Como P Preliminary examination of the comorbidity of anxiety and depression in Parkinson's disease J Nneuropsychiatry Clini Neurosci 1992 4 257 264 10.1176/jnp.4.3.257 1498578

[B264] Vazquez A Jimenez-Jimenez FJ Garcia-Ruiz P Garcia-Urra D "Panic attacks" in Parkinson's disease. A long-term complication of levodopa therapy Acta Neurol Scand 1993 87 14 18 8424307

[B265] Puente V De Fabregues O Oliveras C Ribera G Pont-Sunyer C Vivanco R Cucurella G Giralt E Delgado T Garcia C Seoane A Campo R Eighteen month study of continuous intraduodenal levodopa infusion in patients with advanced Parkinson's disease: impact on control of fluctuations and quality of life Parkinsonism Relat Disord 2010 16 218 221 19762271 10.1016/j.parkreldis.2009.07.015

[B266] Rodrigues JP Walters SE Watson P Stell R Mastaglia FL Globus pallidus stimulation improves both motor and nonmotor aspects of quality of life in advanced Parkinson's disease Mov Disord 2007 22 1866 1870 17659634 10.1002/mds.21427

[B267] Happe S Berger K The association between caregiver burden and sleep disturbances in partners of patients with Parkinson's disease Age Ageing 2002 31 349 354 12242196 10.1093/ageing/31.5.349

[B268] Aarsland D Larsen JP Karlsen K Lim NG Tandberg E Mental symptoms in Parkinson's disease are important contributors to caregiver distress Int J Geriatr Psychiatry 1999 14 866 874 10521886

[B269] Berry RA Murphy JF Well-being of caregivers of spouses with Parkinson's disease Clin Nurs Res 1995 4 373 386 7580943 10.1177/105477389500400404

[B270] O'Reilly F Finnan F Allwright S Smith GD Ben-Shlomo Y The effects of caring for a spouse with Parkinson's disease on social, psychological and physical well-being Br J Gen Pract 1996 46 507 512 8917868 PMC1239744

[B271] McCabe MP Firth L O'Connor E A comparison of mood and quality of life among people with progressive neurological illnesses and their caregivers J Clin Psychol Med Settings 2009 16 355 362 19639395 10.1007/s10880-009-9168-5

[B272] Gallagher D Rose J Rivera P Lovett S Thompson LW Prevalence of depression in family caregivers Gerontologist 1989 29 449 456 2521102 10.1093/geront/29.4.449

[B273] O'Connor EJ McCabe MP Predictors of quality of life in carers for people with a progressive neurological illness: a longitudinal study Qual Life Res 2011 20 703 711 21127997 10.1007/s11136-010-9804-4

[B274] A'Campo LE Spliethoff-Kamminga NG Macht M Roos RA Caregiver education in Parkinson's disease: formative evaluation of a standardized program in seven European countries Qual Life Res 2010 19 55 64 19946755 10.1007/s11136-009-9559-y PMC2804793

[B275] Karlsen KH Tandberg E Arsland D Larsen JP Health related quality of life in Parkinson's disease: a prospective longitudinal study J Neurol Neurosurg Psychiatry 2000 69 584 589 11032608 10.1136/jnnp.69.5.584 PMC1763406

[B276] Maurel F Lilliu H Le Pen C [Social and economic cost of L-DOPA-induced dyskinesias in patients with Parkinson's disease] Rev Neurol (Paris) 2001 157 507 514 11438770

[B277] Suh DC Pahwa R Mallya U Treatment patterns and associated costs with Parkinson's disease levodopa induced dyskinesia J Neurol Sci 2012 319 24 31 22664154 10.1016/j.jns.2012.05.029

[B278] Canadian Institute for Health Information The Burden of Neurological Diseases, Disorders and Injuries in Canada 2007 Ottawa

[B279] Huse DM Schulman K Orsini L Castelli-Haley J Kennedy S Lenhart G Burden of illness in Parkinson's disease Mov Disord 2005 20 1449 1454 16007641 10.1002/mds.20609

[B280] Wang G Cheng Q Zheng R Tan YY Sun XK Zhou HY Ye XL Wang Y Wang Z Sun BM Chen SD Economic burden of Parkinson's disease in a developing country: a retrospective cost analysis in Shanghai, China Mov Disord 2006 21 1439 1443 16773620 10.1002/mds.20999

[B281] Haycox A Armand C Murteira S Cochran J Francois C Cost effectiveness of rasagiline and pramipexole as treatment strategies in early Parkinson's disease in the UK setting: an economic Markov model evaluation Drugs Aging 2009 26 791 801 19728752 10.2165/11316770-000000000-00000

[B282] LePen C Wait S Moutard-Martin F Dujardin M Ziegler M Cost of illness and disease severity in a cohort of French patients with Parkinson's disease PharmacoEconomics 1999 16 59 69 10539122 10.2165/00019053-199916010-00006

[B283] Gottwald MD Aminoff MJ Therapies for dopaminergic-induced dyskinesias in Parkinson disease Ann Neurol 2011 69 919 927 21681795 10.1002/ana.22423

[B284] Olanow CW Watts RL Koller WC An algorithm (decision tree) for the management of Parkinson's disease (2001): treatment guidelines Neurology 2001 56 Suppl 5 S1 S88 11402154 10.1212/wnl.56.suppl_5.s1

[B285] Goetz CG Nutt JG Stebbins GT The Unified Dyskinesia Rating Scale: presentation and clinimetric profile Mov Disord 2008 23 2398 2403 19025759 10.1002/mds.22341

[B286] Goetz CG Stebbins GT Theeuwes A Stocchi F Ferreira JJ van de Witte S Bronzova J Temporal stability of the Unified Dyskinesia Rating Scale Mov Disord 2011 26 2556 2559 21915907 10.1002/mds.23931

[B287] Hagell P Widner H Clinical rating of dyskinesias in Parkinson's disease: use and reliability of a new rating scale Mov Disord 1999 14 448 455 10348468 10.1002/1531-8257(199905)14:3<448::aid-mds1010>3.0.co;2-0

[B288] Colosimo C Martinez-Martin P Fabbrini G Hauser RA Merello M Miyasaki J Poewe W Sampaio C Rascol O Stebbins GT Schrag A Goetz CG Task force report on scales to assess dyskinesia in Parkinson's disease: critique and recommendations Mov Disord 2010 25 1131 1142 20310033 10.1002/mds.23072

[B289] Katzenschlager R Schrag A Evans A Manson A Carroll CB Ottaviani D Lees AJ Hobart J Quantifying the impact of dyskinesias in PD: the PDYS-26: a patient-based outcome measure Neurology 2007 69 555 563 17679674 10.1212/01.wnl.0000266669.18308.af

[B290] Fenney A Jog MS Duval C Bradykinesia is not a "systematic" feature of adult-onset Huntington's disease; implications for basal ganglia pathophysiology Brain Res 2008 1193 67 75 18177845 10.1016/j.brainres.2007.12.005

[B291] Duval C Fenney A Jog MS Groenewegen HJ, Berendse HW, Cools AR, Voorn P, Mulder AB The dynamic relationship between voluntary and involuntary motor behaviors in patients with movement disorders The Basal Ganglia IX 2009 58 Springer; New York 521 534 *Advances in Biology*

[B292] Duval C Panisset M Sadikot AF The relationship between physiological tremor and the performance of rapid alternating movements in healthy elderly subjects Exp Brain Res 2001 139 412 418 11534864 10.1007/s002210100780

[B293] Lemieux S Ghassemi M Jog M Edwards R Duval C The influence of levodopa-induced dyskinesias on manual tracking in patients with Parkinson's disease Exp Brain Res 2007 176 465 475 16944114 10.1007/s00221-006-0632-2

[B294] Duval C Rest and postural tremors in patients with Parkinson's disease Brain Res Bull 2006 70 44 48 16750481 10.1016/j.brainresbull.2005.11.010

[B295] Duval C Sadikot AF Panisset M The detection of tremor during slow alternating movements performed by patients with early Parkinson's disease Exp Brain Res 2004 154 395 398 14666394 10.1007/s00221-003-1676-1

[B296] Ghassemi M Lemieux S Jog M Edwards R Duval C Bradykinesia in patients with Parkinson's disease having levodopa-induced dyskinesias Brain Res Bull 2006 69 512 518 16647580 10.1016/j.brainresbull.2006.02.015

[B297] Duval C Sadikot AF Panisset M Bradykinesia in patients with essential tremor Brain Res 2006 1115 213 216 16920079 10.1016/j.brainres.2006.07.066

[B298] Wierzbicka MM Staude G Wolf W Dengler R Relationship between tremor and the onset of rapid voluntary contraction in Parkinson's disease J Neurol Neurosurg Psychiatry 1993 56 782 787 8331354 10.1136/jnnp.56.7.782 PMC1015060

[B299] Goodman D Kelso JA Exploring the functional significance of physiological tremor: a biospectroscopic approach Exp Brain Res 1983 49 419 431 6641839 10.1007/BF00238783

[B300] Freund HJ Hefter H The role of basal ganglia in rhythmic movement Adv Neurol 1993 60 88 92 8420222

[B301] Fitts PM The information capacity of the human motor system in controlling the amplitude of movement J Exp Psychol 1954 47 381 391 13174710

[B302] Wenzelburger R Peak-dose dyskinesia; an acceptable price for mobility in late-stage Parkinson's disease? Clin Neurophysiol 2005 116 1997 1998 16055380 10.1016/j.clinph.2005.06.009

